# The Sixth Element: a 102-kb RepABC Plasmid of Xenologous Origin Modulates Chromosomal Gene Expression in Dinoroseobacter shibae

**DOI:** 10.1128/msystems.00264-22

**Published:** 2022-08-03

**Authors:** Sonja Koppenhöfer, Jürgen Tomasch, Victoria Ringel, Lukas Birmes, Henner Brinkmann, Cathrin Spröer, Michael Jarek, Hui Wang, Silke Pradella, Irene Wagner-Döbler, Jörn Petersen

**Affiliations:** a Department of Biology, Memorial University of Newfoundland, St. John’s, Newfoundland, Canada; b Laboratory of Anoxygenic Phototrophs, Institute of Microbiology of the Czech Academy of Science—Centre Algatech, Třeboň, Czech Republic; c Leibniz-Institut DSMZ-Deutsche Sammlung von Mikroorganismen und Zellkulturen GmbH, Braunschweig, Germany; d Group Genome Analytics, Helmholtz Centre for Infection Research, Braunschweig, Germany; e Institute of Microbiology, TU Braunschweig, Braunschweig, Germany; University of Hawaii at Manoa

**Keywords:** *Roseobacteraceae*, plasmid stability, transcriptomics, denitrification, heavy metal resistance, CtrA regulon

## Abstract

The model organism Dinoroseobacter shibae and many other marine *Rhodobacterales* (*Roseobacteraceae*, *Alphaproteobacteria*) are characterized by a multipartite genome organization. Here, we show that the original isolate (Dshi-6) contained six extrachromosomal replicons (ECRs), whereas the strain deposited at the DSMZ (Dshi-5) lacked a 102-kb plasmid. To determine the role of the sixth plasmid, we investigated the genomic and physiological differences between the two strains. Therefore, both genomes were (re)sequenced, and gene expression, growth, and substrate utilization were examined. For comparison, we included additional plasmid-cured strains in the analysis. In the Dshi-6 population, the conjugative 102-kb RepABC-9 plasmid was present in only about 50% of the cells, irrespective of its experimentally validated stability. In the presence of the sixth plasmid, copy number changes of other ECRs, in particular, a decrease of the 86-kb plasmid, were observed. The most conspicuous finding was the strong influence of plasmids on chromosomal gene expression, especially the repression of the CtrA regulon and the activation of the denitrification gene cluster. Expression is inversely controlled by either the presence of the 102-kb plasmid or the absence of the 86-kb plasmid. We identified regulatory genes on both plasmids, i.e., a sigma 70 factor and a quorum sensing synthase, that might be responsible for these major changes. The tremendous effects that were probably even underestimated challenge the current understanding of the relevance of volatile plasmids not only for the original host but also for new recipients after conjugation.

**IMPORTANCE** Plasmids are small DNA molecules that replicate independently of the bacterial chromosome. The common view of the role of plasmids is dominated by the accumulation of resistance genes, which is responsible for the antibiotic crisis in health care and livestock breeding. Beyond rapid adaptations to a changing environment, no general relevance for the host cell’s regulome was attributed to these volatile ECRs. The current study shows for the model organism D. shibae that its chromosomal gene expression is strongly influenced by two plasmids. We provide evidence that the gain or loss of plasmids not only results in minor alterations of the genetic repertoire but also can have tremendous effects on bacterial physiology. The central role of some plasmids in the regulatory network of the host could also explain their persistence despite fitness costs, which has been described as the “plasmid paradox.”

## INTRODUCTION

Plasmids are DNA molecules that replicate independently of the chromosome and are one of the major driving forces of prokaryotic evolution via horizontal gene transfer (1). Plasmids are usually transmitted by conjugation, i.e., via direct cell-cell contact between a donor and a recipient, thus enabling the transfer of larger amounts of genetic information than by transduction or transformation (transfer of genetic material via phages or uptake from the environment). Prominent examples are modular resistance gene cassettes against multiple antibiotics, which are especially feared in the hospital environment (2), or the 40-kb photosynthesis gene cluster of *Proteobacteria* (3). Plasmid-borne genes that are not required for replication or maintenance are part of the accessory gene pool that provide new physiological traits, contribute public goods like biofilm components, or can harm neighboring cells, to mention but a few (4, 5). In this way, they have the potential to open up new ecological niches for the host (1).

Plasmids interact with the chromosome of the host cell in complex ways. Transposable elements and integrons mediate intragenomic gene transfer between different replicons, as reported previously for the carbapenem resistance genes in *Enterobacteriaceae* (6, 7). So-called genomic dominance, i.e., the presence of multiple copies of a gene localized on plasmids and the chromosome, might have substantial phenotypic effects on the organism (8, 9). An interplaying regulation of gene expression has recently been documented for two plasmids of the rhizobial strain Agrobacterium tumefaciens 15955. The tumor-inducing plasmid pTi15955 and the megaplasmid pAt15955 carry paralogous quorum sensing (QS) systems that preferentially activate their own *tra* genes for conjugative transfer and cross-activate the *tra* genes of both replicons, respectively (10). Moreover, it has already been found that the uptake of plasmids as single-stranded DNA sequences into the cell has a major influence on chromosomal gene expression via the induction of the bacterial SOS response (11). An active SOS response system increases the mutagenesis rate of the cell and, thus, bacterial evolvability (12, 13).

Carrying a plasmid is energetically costly since the additional DNA needs to be replicated and the genes have to be transcribed (14). Therefore, its stable maintenance should either confer a growth advantage or ensure the survival of the cell in the natural habitat. The frequency of experiencing such conditions in the environment, the strength of the selective pressure, and the energetic costs of maintaining the plasmid determine its prevalence in a population (15, 16). Maintenance, especially of small cryptic plasmids, is ensured by addiction modules such as toxin-antitoxin systems that represent the archetype of selfish genes (17). Without selection pressure, plasmid-free cells are therefore expected to outperform plasmid-carrying cells, and the plasmid becomes extinct (18). However, the plasmid burden, which was initially investigated in carbon-limited chemostat cultures of Escherichia coli (14), might have a much lower or even negligible relevance in nutrient-rich natural habitats because total DNA synthesis in bacteria consumes only about 3% of the cell’s energy budget (19).

*Roseobacteraceae* (syn., *Roseobacter* clade, *Roseobacter* group, or roseobacters) are marine representatives of the order *Rhodobacterales* within the class *Alphaproteobacteria* (20). This group is one of the most abundant and extensively studied bacterial lineages in the ocean and is especially dominant in coastal regions, algal blooms, and, with specific taxa, also polar regions (21, 22). *Roseobacteraceae* genomes are characterized by the high number and diversity of extrachromosomal replicons (ECRs) that have been identified in many members (21). Low-copy-number plasmids with a RepABC-type replication system are the most abundant ECRs of this family (23, 24). Biofilm plasmids with a characteristic rhamnose operon, which were first discovered in Phaeobacter inhibens DSM 17395, mediate the switch between a motile and a sessile lifestyle (25, 26). Further ECRs of *Roseobacteraceae* with an essential role in survival in the environment are flagellar and photosynthesis plasmids and those encoding the synthesis pathways for the antibiotic tropodithietic acid (3, 27).

Dinoroseobacter shibae DFL 12 is one model organism of the *Roseobacteraceae* and was originally isolated from the phycosphere of the dinoflagellate Prorocentrum lima (28). It has been well investigated for its quorum sensing system (29), gene transfer agent (30), metabolic adaptations (31, 32), and interaction with the algal host (28, 33–35). The genome of D. shibae comprises five ECRs: two chromids (153 and 72 kb) with chromosome-like GC content and codon usage and three genuine plasmids (191, 126, and 86 kb) showing a largely deviating genomic imprint (23, 27). The two syntenous RepABC-type plasmids of 191 and 126 kb in size, which have been classified as RepABC-9 and RepABC-2 (28), contain characteristic type IV secretion systems (T4SSs) that mediate their conjugational transfer (18, 23). The expression levels of these T4SSs increased 2- to 3-fold when a quorum sensing-deficient strain was complemented by the external addition of *N*-acyl-homoserine lactones (AHLs) (33). In cocultivation experiments with the dinoflagellate Prorocentrum minimum, the 191-kb plasmid was found to be responsible for algal killing (34, 35). The three remaining ECRs of 153, 86, and 72 kb in size were classified according to their replication systems as RepA-I-, RepABC-1-, and RepB-I-type replicons (23). The 153-kb chromid plays an essential role, at least under salt shock conditions (36); the 86-kb plasmid affects energy production and harbors one of three quorum sensing synthases (29); and the 72-kb chromid is crucial for survival during starvation and oxidative stress (37). The SOS system, in combination with quorum sensing and cell cycle control, is part of an integrated transcriptional regulatory network (38).

The type strain of *D. shibae* was deposited at the DSMZ (German Collection of Microorganisms and Cell Cultures) more than 15 years ago as DSM 16493^T^ (DFL 12^T^). However, pulsed-field gel electrophoresis (PFGE) showed that the deposited and genome-sequenced strain with five ECRs (28) has lost a sixth replicon with a size of 102 kb that was present in the original isolate (39). Meanwhile, this DFL 12^T^ isolate with six ECRs has been deposited at the DSMZ as strain DSM 112351. For the sake of brevity, *D. shibae* strain DSM 16493^T^ with five ECRs is designated Dshi-5, and strain DSM 112351 with six ECRs is named Dshi-6.

Here, we completely (re)sequenced the genomes of Dshi-5 and Dshi-6 to identify possible additional genetic differences between the two deposited genetic variants of DFL 12^T^. We then studied the physiological role of the 102-kb plasmid in *D. shibae*, namely, its prevalence in the population, gene expression, and effect on growth, heavy metal tolerance, and substrate utilization. By comparing the transcriptomes of Dshi-5 and Dshi-6, we found that the sixth element had a huge impact on chromosomal gene expression. Therefore, we asked if this finding was specific to the 102-kb plasmid or would also occur upon the loss of other ECRs. By analyzing the transcriptomes of Dshi-5 mutants cured of the 191-kb (killer) plasmid, the 86-kb (quorum sensing) plasmid, and the 72-kb (starvation) chromid in comparison to wild-type Dshi-5, we found both specific and shared responses to the loss of the ECRs.

## RESULTS

### Genome sequencing of Dinoroseobacter shibae DFL 12^T^.

**FIG 1:**
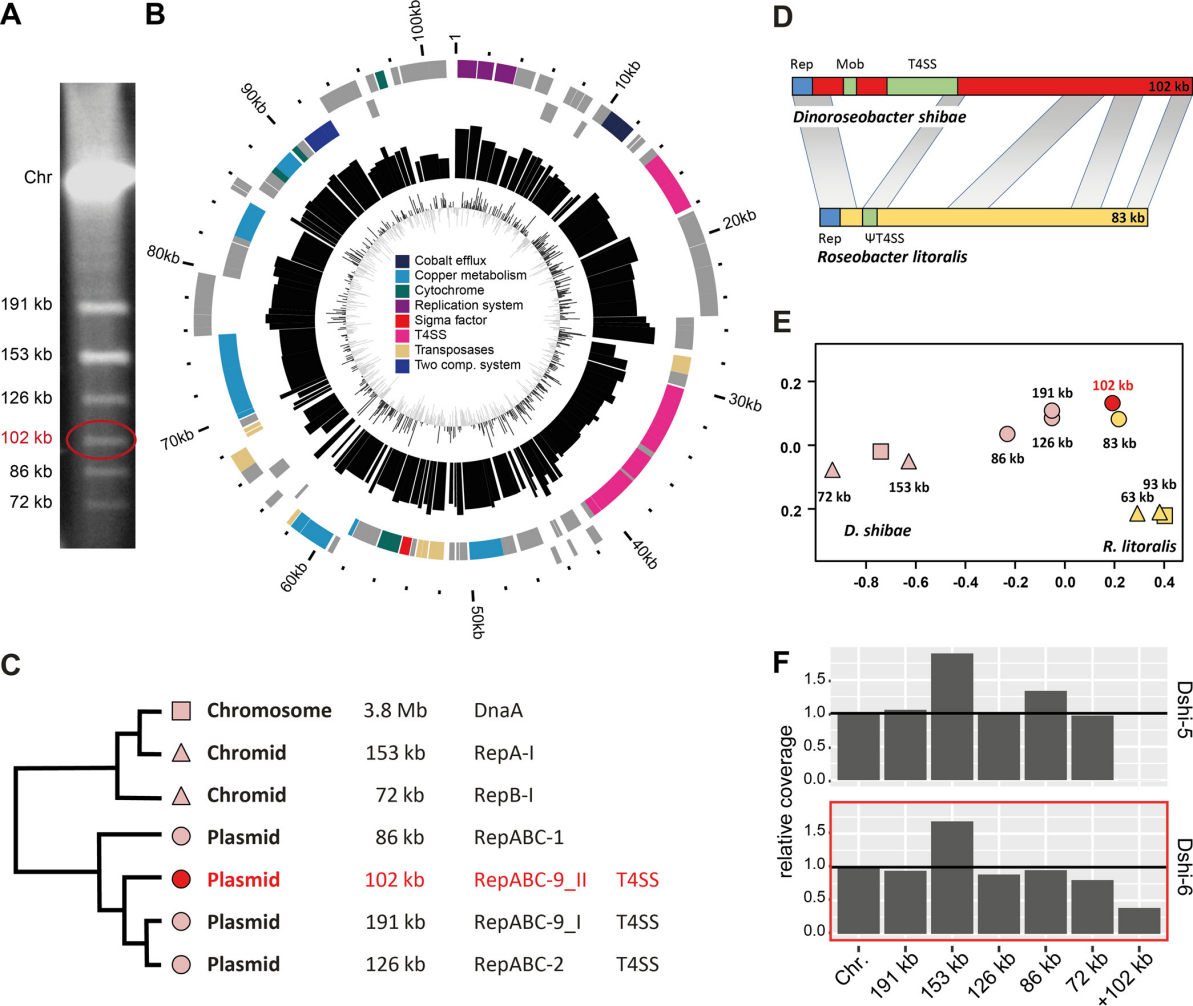
The 102-kb plasmid of *D. shibae*. (A) Pulsed-field gel electrophoresis of strain Dshi-6. (B) Circular visualization of the 102-kb plasmid, with major gene groups indicated. The plasmid was oriented based on its RepABC-9_II-type replication system. The outer to inner rings show (i) the scale of plasmid size in kilobases; (ii) the locations of gene groups on the plus and minus strands, where groups were identified based on automatic and manual annotations; (iii) gene expression via bar plots of the log reads per kilobase per million mapped reads (RPKM) with a scale of 5 to 19; and (iv) GC skew. (C) Classification of chromids and plasmids based on relative synonymous codon usage (RSCU) analysis. (D) Synteny plot of the 102-kb plasmid from *D. shibae* and the 86-kb plasmid from Roseobacter litoralis. *Mob*, *virD*-type mobilization genes. (E) RSCU analysis of Dshi-6 and *R. litoralis*. (F) Coverage of all replicons in Dshi-5 and Dshi-6. Coverage is shown relative to the chromosome with 1 equivalent per cell.

The genome-sequenced type strain of Dinoroseobacter shibae, DFL 12^T^ (DSM 16493^T^) (Dshi-5), contains five extrachromosomal replicons (ECRs) (28). However, pulsed-field gel electrophoresis (PFGE) analyses of the original *D. shibae* DFL 12^T^ glycerol stock (Dshi-6) surprisingly showed the presence of a sixth plasmid with a size of 102 kb (Fig. 1A), which was lost during preservation and deposition in the public culture collection.

We isolated, sequenced, and annotated the additional 102-kb plasmid, which is available under BioProject accession number PRJNA814221. (Re)sequencing of the complete genomes of both *D. shibae* strains (Dshi-5 [GenBank assembly accession number GCA_000018145.1] and Dshi-6 [accession number GCA_023558335.1]) documented their identity, except for the loss of the sixth element and the presence of one single-nucleotide polymorphism (SNP) on the chromosome of Dshi-5 (see Table S1B in the supplemental material). This SNP is located in the 30S ribosomal protein S1 gene (RpsA [Dshi_1025]) of Dshi-6 and leads to an amino acid exchange from glutamic acid to lysine (E205K). A phylogenetic comparison with 20 closely related *Roseobacteraceae* showed that lysine represents the prevalent amino acid at RpsA protein position 205 (Table S1C). The strain with six ECRs was hence deposited at the DSMZ to allow future comparisons of the original Dshi-6 isolate (DSM 112351) to the spontaneously cured mutant isolate Dshi-5 (DSM 16493^T^).

10.1128/msystems.00264-22.10 TABLE S1 (A) Complete data set of all strains used in this study. (B) Comparison of the Dinoroseobacter shibae DSM 16493^T^ reference genome sequence with the sequences of the resequenced Dshi-5 strain (DSM 16493^T^) and Dshi-6 (DSM 112351). (C) Amino acid alignment of the 30S ribosomal protein S1 (RpsA [Dshi_1025]). Mutations of the original glutamic acid (E) of Dshi-5 at position 205 are highlighted in red. Conserved amino acids are indicated by a dot. (D) GenBank accession numbers for RepA, RepB, and RepC proteins of RepABC-9-type plasmid replication systems used for phylogenetic analyses. (E) Homologous proteins shared by the six extrachromosomal replicons of Dinoroseobacter shibae DSM 112351. (F) Homologous proteins of *P. inhibens* P88 plasmid pP88_b, Roseobacter litoralis DSM 6996^T^ plasmid pRLO149_83, and the 102-kb plasmid. Download Table S1, XLSX file, 0.7 MB. Copyright © 2022 Koppenhöfer et al. 2022 Koppenhöfer et al. https://creativecommons.org/licenses/by/4.0/ This content is distributed under the terms of the Creative Commons Attribution 4.0 International license.

Finally, we sequenced the complete genome of *D. shibae* DFL-36 (GenBank assembly accession number GCA_023558355.1), which was isolated from another dinoflagellate culture (40). This reference strain also comprises the 102-kb plasmid, and its genome organization with six ECRs is identical to that of Dshi-6.

### The 102-kb plasmid of Dinoroseobacter shibae Dshi-6 (DSM 112351).

The sixth ECR of *D. shibae* comprises a total of 116 protein-encoding genes (Fig. 1B). Relative synonymous codon usage (RSCU) analysis of the complete *D. shibae* genome (23) showed that the additional 102-kb replicon represents a genuine plasmid (Fig. 1C). This assessment is in agreement with the presence of a characteristic type IV secretion system (T4SS), including the *virD2D4* mobilization operon for plasmid conjugation. The functionality of homologous T4SSs from the 191- and 126-kb sister plasmids has been experimentally validated (33, 41). The presence of a two-component system (Dshi_6153-Dshi_6154) and a sigma 70 factor (Dshi_6114) indicates the potential regulatory interference of the 102-kb plasmid with the gene expression of the host genome.

The 102-kb plasmid contains a characteristic RepABC-type replication system that ensures its maintenance with 1 or 2 copies per cell (24). Phylogenetic analysis of the replicase RepC (Dshi_6053) showed that two replicons of *D. shibae* (102 kb and 191 kb) represent RepABC-9-type plasmids (Fig. S1A and Table S1D). A comparable observation was made for two RepC proteins from Marinovum algicola DSM 10251^T^ that are also located in the RepC-9 subtree. These findings were surprising because the phylogenetic position of plasmid replication proteins (and their adjacent partitioning) in distinct subtrees is generally regarded as a diagnostic trait for the identification of compatibility groups in *Rhodobacterales* (24, 42). Accordingly, we analyzed the RepA, RepB, and RepC proteins of 26 RepABC-9 modules from *Roseobacteraceae* separately with their closest rhizobial relatives (Fig. S1A and Table S1D). The phylogenies clearly documented the synchronous evolution of the RepA and RepB partitioning proteins, in contrast to the replicase RepC, which showed a scattered distribution. The *parAB* partitioning modules of RepABC-9 plasmids were recruited twice from rhizobia (*Hyphomicrobiales*), and the localization of M. algicola DSM 10251^T^ in two RepA/B-9_I subtrees, which are color-coded in blue and green in Fig. S1A, is a strong indicator of different compatibility groups. This conclusion is supported by the presence of diagnostic palindromes that serve as specific *cis*-acting anchors for RepB-mediated plasmid partitioning (Fig. S1B) (24). Accordingly, the current study revealed the presence of five different compatibility groups for RepABC-9-type plasmids from *Rhodobacterales*.

10.1128/msystems.00264-22.1 FIG S1 (A) Phylogenetic RepA, RepB, and RepC analyses of RepABC-9-type plasmid replication modules of *Rhodobacterales*. The complete set of RepC-9 replicase sequences and the adjacent RepA and RepB partitioning genes have been analyzed with rhizobial outgroup sequences (see Table S1D in the supplemental material). The color-code indicates putative compatibility groups of RepABC-9-type replication modules. Blue, RepABC-9_I; orange, A4-B4-C9; green, A12-B12-C9; red, RepABC-9_IIa; pink, RepABC-9_IIb. RepA, RepB, and RepC-9 phylogenies are presented with bright gray and yellow backgrounds, respectively. Taxa with two RepABC-9-type plasmids are shown in boldface type. Dark gray boxes highlight the synchronous evolution of the RepA, RepB, and RepC-9 proteins. ●, 100% bootstrap support. (B) Consensus palindrome sequences of RepABC-9-type plasmids according to phylogenetic analyses (Fig. S1A). Download FIG S1, DOCX file, 1.1 MB. Copyright © 2022 Koppenhöfer et al. 2022 Koppenhöfer et al. https://creativecommons.org/licenses/by/4.0/ This content is distributed under the terms of the Creative Commons Attribution 4.0 International license.

All genes of the 102-kb plasmid are expressed (Fig. 1B). However, a comparison of the average transcription levels of the chromosome and ECRs in Dshi-6 clearly showed that the expression level of the 102-kb plasmid is about 50% lower than those of the other ECRs and the chromosome (Fig. S2), most likely as a result of the presence of this ECR in only 50% of the cells (see below). Interestingly, this ECR contains a high number of genes related to heavy metal resistance that were annotated as genes for copper and cobalt transport systems. However, no differences were observed in growth experiments comparing the copper, cobalt, and zinc resistance of strains Dshi-5 and Dshi-6, while the latter showed slightly increased resistance to silver nitrate (Fig. S3).

10.1128/msystems.00264-22.2 FIG S2 Comparison of the gene expression levels of all seven replicons from Dshi-6. The box plots for three biological replicates (rep1, rep2, and rep3) are shown on a logarithmic scale. Median expression values of the replicons from replicate 1 are as follows: 2,206× for the chromosome (GenBank accession number NC_009952.1), 1,713× for pDSHI01 (accession number NC_009955.1), 1,228× for pDSHI02 (accession number NC_009956.1), 1,740× for pDSHI03 (accession number NC_009957.1), 2,529× for pDSHI04 (accession number NC_009958.1), 1,008× for pDSHI05 (accession number NC_009959.1), and 501× for pDSHI06 (accession number CP097546.1). Download FIG S2, DOCX file, 0.1 MB. Copyright © 2022 Koppenhöfer et al. 2022 Koppenhöfer et al. https://creativecommons.org/licenses/by/4.0/ This content is distributed under the terms of the Creative Commons Attribution 4.0 International license.

10.1128/msystems.00264-22.3 FIG S3 Growth kinetics of strains Dshi-5 (five ECRs) and Dshi-6 (six ECRs) in artificial seawater (ASW) medium with succinate and different heavy metals. Growth was monitored over 50 h and measured as the optical density at 600 nm. Download FIG S3, DOCX file, 0.3 MB. Copyright © 2022 Koppenhöfer et al. 2022 Koppenhöfer et al. https://creativecommons.org/licenses/by/4.0/ This content is distributed under the terms of the Creative Commons Attribution 4.0 International license.

### Homologs of the sixth element.

The 102-, 86-, 191-, and 126-kb plasmids share homologous regions that comprise transposase and integrase genes (Table S1E and Fig. S4A). The 102-kb plasmid also shares conserved regions with plasmids from other *Roseobacteraceae* (Table S1F and Fig. S4B). The closest relative of the sixth element is the syntenous 83-kb plasmid pRLO149_83 from Roseobacter litoralis DSM 6996^T^, with a query coverage of 51% and a sequence identity of up to 98% (Fig. 1D). This plasmid harbors a homologous RepABC-9_II replication cassette (Dshi_6051-Dshi_6053) but lost the capacity for conjugation due to a deletion within the structurally conserved T4SS (missing proteins are VirD2, VirD4 [Dshi_6068-Dshi_6069], VirB11, VirB10, VirB9, VirB8, VirB6, VirB5, and lytic glycosylase [Dshi_6080-Dshi_6088]).

10.1128/msystems.00264-22.4 FIG S4 (A) Sequence alignment of the 102-kb plasmid with three other *D. shibae* replicons. (B) Mauve alignment of the 102-kb plasmid (Dshi-6) with four *Roseobacteraceae*. Download FIG S4, DOCX file, 0.4 MB. Copyright © 2022 Koppenhöfer et al. 2022 Koppenhöfer et al. https://creativecommons.org/licenses/by/4.0/ This content is distributed under the terms of the Creative Commons Attribution 4.0 International license.

The RSCU analysis of all *D. shibae* and R. litoralis replicons also documents the common origin of the syntenous 102-kb and 83-kb plasmids, but moreover, it shows the great distance from their respective chromosomes (Fig. 1E). This genomic imprint reflects the xenologous origin of both RepABC-9_II plasmids, and it suggests comparably late recruitment by their current host.

### Copy number and population prevalence of ECRs.

We then compared the read coverage of the ECRs obtained from DNA sequencing to that of the chromosome to determine their copy numbers (Fig. 1F). In the Dshi-5 reference strain, most ECRs have the same coverage as the chromosome (191 kb, 126 kb, and 72 kb). Therefore, they can be considered single-copy replicons. The coverage of the 86-kb plasmid is increased by about 30%, and the one of the 153-kb chromid is almost doubled. In contrast, the copy numbers of all ECRs are slightly reduced in the Dshi-6 strain, most notably that of the 86-kb plasmid. Examination of the influence of other ECRs showed that experimental curing of the 191-kb and 86-kb plasmids also reduced the coverage of the 86-kb plasmid and the 72-kb chromid, respectively (Fig. S5A and B). Remarkably, the relative abundance of the 102-kb plasmid is <50% compared to the chromosome. These results suggest that the 102-kb plasmid might be comparably unstable and becomes lost during cultivation.

10.1128/msystems.00264-22.5 FIG S5 (A) Coverage of all replicons in each strain used in the study. (B) Coverage of *D. shibae* replicons in different wild-type and curing strains. Download FIG S5, DOCX file, 0.7 MB. Copyright © 2022 Koppenhöfer et al. 2022 Koppenhöfer et al. https://creativecommons.org/licenses/by/4.0/ This content is distributed under the terms of the Creative Commons Attribution 4.0 International license.

In order to investigate the stability of the sixth element, we randomly mutagenized Dshi-6 with the mariner transposon, investigated the insertion sites of 384 gentamicin-resistant colonies, and finally identified a single transposon mutant that tagged the 102-kb plasmid (mutant Dshi-6::Gm_102kb, position 9188 [plus strand]; gene Dshi_6060) (Fig. S6A). Three stability assays of this mutant showed that 99.6% (448/450) of the tested colonies still contained the 102-kb plasmid after at least 7 days of continuous cultivation in either marine broth (MB) or saltwater medium (SWM) (Fig. S6B to D). These findings demonstrate that the 102-kb plasmid of *D. shibae* is stable in both media, but they also show that spontaneous losses of this ECR occasionally occur.

10.1128/msystems.00264-22.6 FIG S6 Transposon mutant and stability tests of the 102-kb strain. Download FIG S6, DOCX file, 1.6 MB. Copyright © 2022 Koppenhöfer et al. 2022 Koppenhöfer et al. https://creativecommons.org/licenses/by/4.0/ This content is distributed under the terms of the Creative Commons Attribution 4.0 International license.

Next, we examined the distribution of the 102-kb replicon in the original isolate Dshi-6 (*D. shibae* DSM 112351) to better resolve the contradiction between its experimentally confirmed stability and its low abundance compared to the chromosome. Investigation of a total of 70 colonies with specific duplex PCR targeting the 102-kb (test) and 191-kb (positive-control) plasmids showed that 35 of the 70 tested colonies lacked the 102-kb plasmid, whereas the 191-kb plasmid was present in all colonies (Fig. S7). This outcome reflects the genetic heterogeneity of the Dshi-6 population with respect to the 102-kb plasmid. Moreover, the very tight copy number control of RepABC-type replicons, where all compatible RepABC-1 (86-kb), RepABC-2 (126-kb), and RepABC-9_I (191-kb) plasmids of *D. shibae* are present in a single copy per cell (Fig. S5B), allowed us to explain the coverage anomaly. The absence of the sixth element in half of the population is thus in accordance with the roughly 50% prevalence of this plasmid in the genome sequencing data.

10.1128/msystems.00264-22.7 FIG S7 Duplex PCR of 70 *D. shibae* DSM 112351 (Dshi-6) colonies for the detection of the 102-kb plasmid and the 191-kb plasmid. Download FIG S7, DOCX file, 1.0 MB. Copyright © 2022 Koppenhöfer et al. 2022 Koppenhöfer et al. https://creativecommons.org/licenses/by/4.0/ This content is distributed under the terms of the Creative Commons Attribution 4.0 International license.

### Influence of different plasmids and chromids on growth.

We investigated the growth of five *D. shibae* strains with different ECR profiles in MB and SWM (Fig. S8). Dshi-5 and Dshi-6 showed nearly identical curves in SWM and similar curves in MB medium, which clearly documented that the loss of the 102-kb plasmid in Dshi-5 did not influence the growth behavior. A similar result with indistinguishable growth curves was obtained for the comparison of Dshi-5 and Δ72kb. In contrast, strain Δ86kb exhibited a conspicuous decline in the optical density at 600 nm (OD_600_) after 25 h of cultivation in MB medium (Fig. S2), whereas Δ191kb showed an increased lag phase in SWM and reached a higher maximum OD_600_ in MB medium. Accordingly, our comparison shows that neither the 72-kb chromid nor the 102-kb plasmid had a major influence on the growth of *D. shibae* under the tested conditions. In contrast, plasmid curing of the 86-kb and 191-kb plasmids resulted in different growth performances of the mutants. Finally, the comparison between SWM and MB medium showed that all five strains grew much better in nutrient-rich MB medium.

10.1128/msystems.00264-22.8 FIG S8 Growth kinetics of strains Dshi-5 (five ECRs), Dshi-6 (six ECRs), and three curing mutants of Dinoroseobacter shibae (Δ72kb, Δ86kb, and Δ191kb) (four ECRs) in different media. Strains were grown in either marine broth (MB) or artificial seawater (ASW) medium with succinate. Growth was monitored over 50 h and measured as the optical density at 600 nm. Download FIG S8, DOCX file, 0.3 MB. Copyright © 2022 Koppenhöfer et al. 2022 Koppenhöfer et al. https://creativecommons.org/licenses/by/4.0/ This content is distributed under the terms of the Creative Commons Attribution 4.0 International license.

### The 102-kb plasmid modulates chromosomal gene expression.

**FIG 2:**
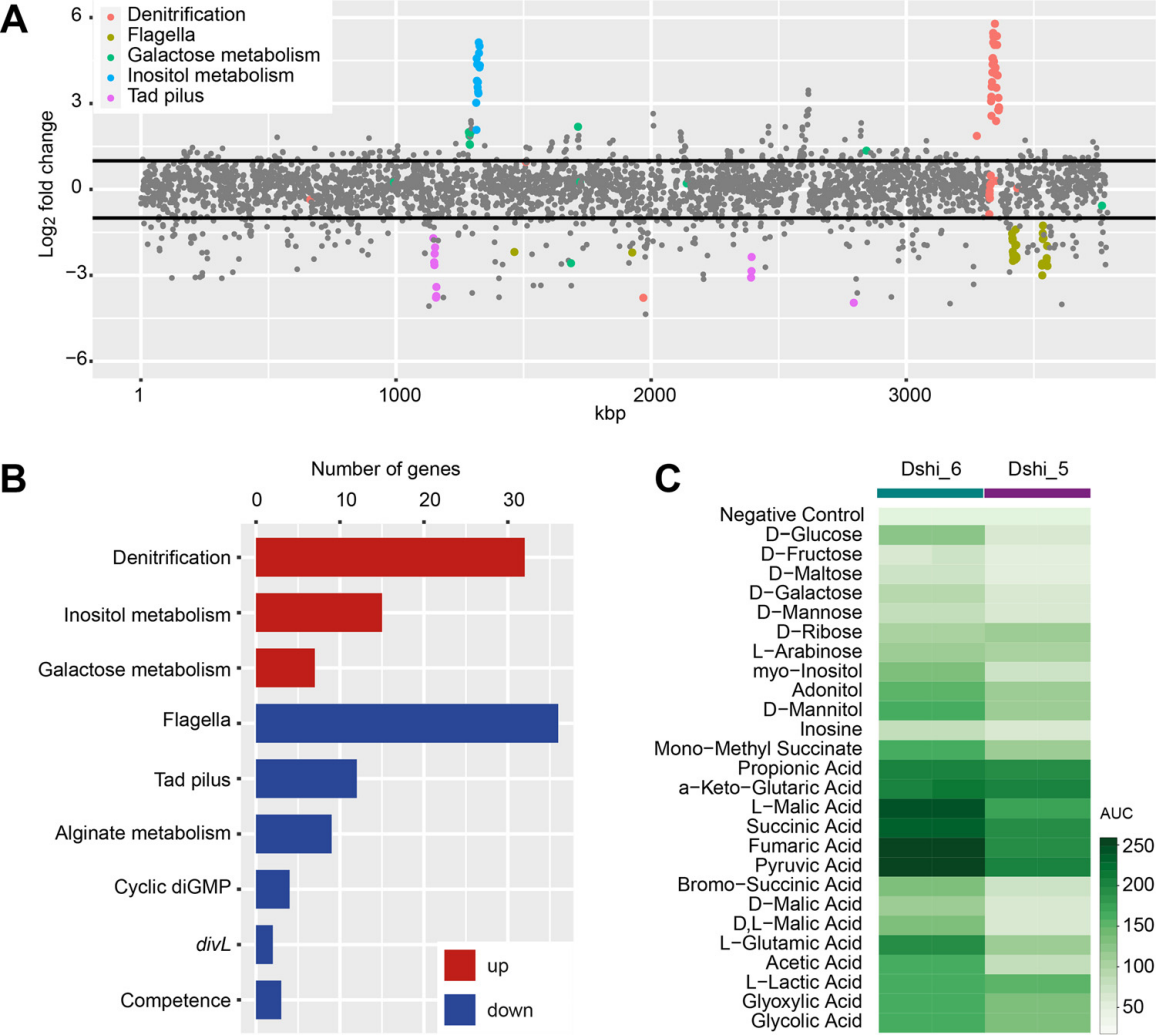
Chromosomal gene expression and phenotypic microarray of Dshi-6 and Dshi-5 reflect the regulatory role of the 102-kb plasmid in *D. shibae*. (A) Differential gene expression of Dshi-6 with the respective location on the chromosome. Strongly regulated clusters are color-coded and labeled. (B) Categories of up- and downregulated genes in Dshi-6. (C) Differential conversion of metabolites. AUC, area under the curve.

The expression of multiple genes was affected by the presence of the 102-kb plasmid (Fig. 2). This included the upregulation of inositol and galactose metabolism and denitrification clusters as well as the downregulation of flagellar, *tad* pilus, alginate metabolism, and cyclic di-GMP genes and the cell cycle regulator DivL (Fig. 2B). In agreement with the results of the transcriptome analysis, the phenotypic microarray showed that myoinositol is mainly converted by Dshi-6 (Fig. 2C; Fig. S9A). The transcriptomic and phenotypic comparisons of Dshi-6 and Dshi-5 showed that further components of sugar metabolism, such as galactose and glucose, were upregulated and metabolized to a greater extent by Dshi-6. In general, *D. shibae* utilizes organic acids like propionic, glutaric, malic, succinic, fumaric, and pyruvic acids better when the 102-kb plasmid is present. The usage of these substrates by the algal symbiont *D. shibae* is in agreement with the reported preference of alga-associated bacteria for organic acids (43).

10.1128/msystems.00264-22.9 FIG S9 (A) Biolog phenotypic microarray (PM1) comparison showing the conversion of 95 carbon sources by the Dshi-6 and Dshi-5 strains. (B) Multidimensional scaling of the transcriptomic data sets analyzed in this study. (C) Venn diagram showing the number of extrachromosomal genes overlappingly regulated in each mutant strain (Dshi-5 and Dshi-6). (D) Correlation of log_2_ fold change values of all genes in the different strains used in this study compared to the reference strain Dshi-5. (E) Enrichment of KEGG pathways in Dshi_6, Δ86kb, and Δ191kb against the reference strain. Download FIG S9, DOCX file, 1.0 MB. Copyright © 2022 Koppenhöfer et al. 2022 Koppenhöfer et al. https://creativecommons.org/licenses/by/4.0/ This content is distributed under the terms of the Creative Commons Attribution 4.0 International license.

### Opposing influences of the 86-kb and 102-kb plasmids on the chromosomal gene expression of *D. shibae*.

To further investigate the influence of the 102-kb plasmid on the gene expression of the chromosome and other replicons, we compared *D. shibae* strain Dshi-6 with the reference strain Dshi-5. We further included three ECR-cured mutant strains of Dshi-5 (Δ191kb, Δ86kb [32], and Δ72kb [37]) in the analysis and compared them with the wild-type Dshi-5 strain to investigate the regulatory role of the 191-kb and 86-kb plasmids as well as the 72-kb chromid. The five strains were first assessed for similarity of their gene expression profiles. Multidimensional scaling (MDS) revealed three groups (Fig. S9B). Dimension 1 clearly separated strains Δ86kb and Dshi-6 from strains Δ191kb, Δ72kb, and Dshi-5. The second dimension separated strain Δ191kb from Dshi-6, Δ72kb, and Dshi-5. We did not further consider the chromid-cured Δ72kb mutant as only one gene (Dshi_3135) was differentially expressed compared to the reference strain.

**FIG 3:**
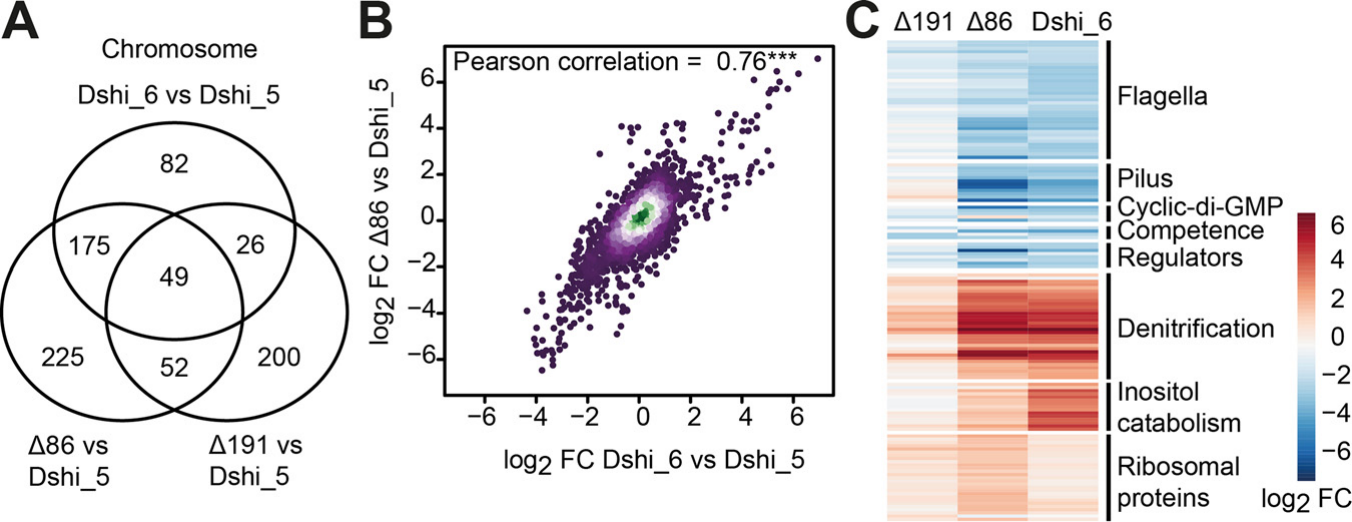
Gene expression of *D. shibae* strains with four and six ECRs (Δ86kb, Δ191kb, and Dshi-6) in comparison with the model organism DSM 16493 (Dshi-5) harboring five ECRs. (A) Venn diagram representing the overlap of significantly (*P* ≤ 0.05) regulated chromosomal genes in three strains compared to the reference strain. (B) Correlation of log_2_ fold change (FC) values of all genes in response to the loss of the 86-kb plasmid or the “gain” of the 102-kb plasmid compared to the reference strain. (C) Heatmap of differentially expressed genes on the *D. shibae* chromosome. Each gene is significantly expressed in at least one of the four strains. Regulators include the partner-switching system *rsbV*-*rsbW* (Dshi_0072-Dshi_0073); Dshi_1507; a stress response regulator, *lexA* (Dshi_1803); two *divL* genes (Dshi_3433-Dshi_3346); and *gafA* (Dshi_1584) with its adjacent gene Dshi_1585.

A Venn diagram shows the overlap of significantly regulated chromosomal genes between Δ191kb, Δ86kb, and Dshi-6, all compared with Dshi-5 (Fig. 3A; for ECR genes, see Fig. S9C). We observed that more than 20% of the chromosomal genes (809/3,668) were differentially regulated by the plasmids, and all three strains shared a common set of 49 differentially regulated genes. These include genes of the denitrification (Dshi_3160-Dshi_3199), flagellar (Dshi_3246-Dshi_3267, Dshi_3358-Dshi_3365, Dshi_3376-Dshi_3380, Dshi_1409, and Dshi_1845) and alginate (Dshi_1895-Dshi_1904) gene clusters as well as some individual genes (Table S1A). However, the majority of the 507 genes in total were controlled by only one strain (82 [Dshi-6], 200 [Δ191kb], and 225 [Δ86kb] genes), with the cured mutant lacking the 86-kb plasmid controlling the largest regulon without overlapping another mutant (Fig. 3A). With 175 plus 49 differentially regulated genes, the Δ86kb and Dshi-6 strains showed the largest overlap.

Little impact of each mutant was found on the gene expression of the remaining extrachromosomal replicons (Table S1A). No genes on other ECRs were significantly regulated in response to the loss of the 72-kb chromid, while the loss of the 86-kb plasmid affected the gene expression of components of the NitT/TauT family transport system (Dshi_4185-Dshi_4189 [72-kb chromid]), hemolysin (Dshi_3871-Dshi_3872 [153-kb chromid]), and an *imuA-imuB-dnaE2*-type mutagenic cassette (Dshi_3995-Dshi_3998 [126-kb plasmid]). In strain Δ191kb, the NitT/TauT family transport system is also regulated beyond a TRAP transporter (Dshi_4202-Dshi_4205 [72-kb plasmid]) and a couple of genes in two clusters on the 126-kb plasmid (Dshi_3936-Dshi_3948 and Dshi_4027-Dshi_4049).

Due to the large number of shared genes differentially expressed in Δ86kb and Dshi-6 compared to the reference strain Dshi-5, we determined the correlation between the two strains, taking all genes into account (Fig. 3B). The highly significant Pearson correlation coefficient of 0.76 between Δ86kb and Dshi-6 demonstrates the high similarity between both strains that exceeds a simple overlap in significantly regulated genes. We did not observe such a high correlation with any other strain combination (Fig. S9D).

### Regulation of the denitrification and CtrA regulons by the 86-kb and 102-kb plasmids.

Among the most strongly upregulated gene clusters in the Δ86kb and Dshi-6 strains were those for denitrification (Fig. 3C; Table S1A and Fig. S9E). There are three denitrification gene clusters for the nitrite reductase (Nir), nitric oxide reductase (Nor), and nitrous oxide reductase (Nos) that were activated in both strains, while the fourth gene cluster for the nitrate reductase (Nap) showed only a small change in expression, which is consistent with the unique regulatory pattern previously observed for *nap* genes (31, 44). Among the aerobe/anaerobe transcriptional regulators, *dnrE* (Dshi_3191) was the only one that was activated in the Δ86kb and Dshi-6 strains. No effect was observed for *fnrL* (Dshi_0660) and *dnrF-dnrD* (Dshi_3270-Dshi_3189).

In contrast, the most downregulated genes in both strains were part of the CtrA regulon (38, 45). These included the flagellar gene cluster, the tight adherence (Tad) pilus (46), and all cyclic di-GMP and competence genes. Among the cyclic di-GMP genes, diguanylate cyclase 2 was the only activated and phosphodiesterase 1 was the most downregulated gene in Dshi-6, and the same but even more pronounced regulation was observed in the Δ86kb strain. In contrast to the regulon, the regulatory genes themselves, consisting of the CtrA phosphorelay and quorum sensing components, were not affected by the presence of the 102-kb plasmid. Unlike the remaining CtrA regulon, only the gene transfer agent (GTA) gene cluster was slightly increased in the Δ86kb and Dshi-6 strains. Interestingly, the large terminase (Dshi_2178) and *gafA* (Dshi_1584), which was suggested previously to control the proportion of the GTA-producing subpopulation (38, 47), were highly expressed in both strains.

## DISCUSSION

### The coverage anomaly of the sixth element: high stability and no metabolic burden of the 102-kb plasmid.

Genome sequencing of *D. shibae* DFL 12 confirmed the presence of a sixth, 102-kb plasmid in the original isolate Dshi-6 in addition to the five ECRs that are found in the deposited reference strain Dshi-5 (28). Mapping of the sequence reads on all replicons of strain Dshi-6 indicated that it represents a heterogeneous population with respect to the sixth element, and this prediction was validated by the investigation of single colonies that documented the absence of the 102-kb plasmid in 50% of the cells. Accordingly, the effects of the sixth element on the physiology of *D. shibae*, especially gene regulation, have probably been underestimated. We showed that the observed distribution is caused neither by plasmid instability nor by growth differences. The stability assays of the 102-kb RepABC-9_II plasmid documented its conspicuous durability. The genuine gentamicin-tagged plasmid was still present in 99.6% of the tested colonies after 1 week of continuous cultivation in nutrient-rich medium without any selection (see Fig. S6 in the supplemental material), whereas cloned RepABC modules from rhizobia and *Rhodobacterales* have been shown to be lost in 2.5% to 90% of the host cells after cultivation overnight (48). Furthermore, the comparison of Dshi-5 and Dshi-6 revealed nearly identical growth curves in SWM, and it even indicated a slightly better growth performance of Dshi-6 in MB medium (Fig. S1), which would not result in successive outcompeting of the sixth element under standard cultivation conditions. The comparable growth is *a priori* counterintuitive due to the energetic costs of the replication and maintenance of an ECR (49), which is designated the plasmid burden (50, 51), but it might be explained by the pivotal role of the 102-kb plasmid in the regulation of host cell gene expression (Fig. 2). Based on the genomic proportion of the sixth element (2%) and the energetic costs of DNA synthesis (3%) (19), the replication of the single-copy-number 102-kb plasmid accounts for less than 0.1% of the total energy budget of *D. shibae*. The stable integration of plasmids into the sophisticated regulatory network of bacteria would thus overcompensate for the minuscule energetic costs for their maintenance, in analogy to the overhead for administrative and controlling units of companies.

### The RepABC-9 replication system of the 102-kb plasmid.

The coexistence of low-copy-number ECRs in the same organism serves as a unique criterion for the identification of different compatibility groups (24, 42). Complete genome sequencing of Dshi-6 revealed the presence of four single-copy RepABC-type plasmids, which hence represent individual compatibility groups. Two of them were classified as RepABC-1 and RepABC-2 plasmids (86 kb and 126 kb), but surprisingly, we found that the 191- and 102-kb plasmids of *D. shibae* both represent the replicon type RepABC-9. RepABC-1 to RepABC-8 plasmids of *Rhodobacterales* have a common origin and can be unequivocally classified based on their synchronously evolving RepA, RepB, and RepC proteins and a diagnostic palindrome (24). In contrast, RepABC-9 plasmids of *Rhodobacterales* originated independently from a rhizobial progenitor, and their replication modules still exhibit a feature characteristic of rhizobia (48), i.e., the synchronous evolution of the *repAB* operon, including the palindrome, combined with frequent exchanges of the *repC* gene (Fig. S1A and B). Our comparative analyses showed that the “rhizobium-like” RepABC-9-type plasmids of *Rhodobacterales* are represented by five compatibility groups that are phylogenetically distinguishable.

RepABC is the most abundant replicon type in rhizobia, and the characteristic tripartite modules are responsible for the reliable replication and partitioning of plasmids, chromids, megaplasmids, and even secondary chromosomes (52–54). The wealth of up to six RepABC-type plasmids that has been reported for the symbiotic nitrogen-fixing organism Rhizobium etli CFN42 (55) documents the presence of many compatibility groups. However, plasmid incompatibility in rhizobia is poorly understood, and previous analyses were focused mainly on RepABC systems of the same host (56). The phylogenetic classification of RepABC-type plasmids, which should, according to the insights from the current study, be based exclusively on their partitioning proteins (RepA and RepB), provides a promising perspective to understand the multipartite genome organization of rhizobia.

### Antagonistic regulation of the chromosomally localized CtrA regulon by two plasmids.

The presence of the 102-kb plasmid activates the denitrification gene clusters and inhibits parts of the CtrA regulon, including the flagellar gene clusters. This raises the question of how the signal from the plasmid is integrated into this regulation. When the CtrA phosphorelay is active, its own genes are also regulated differentially as well, due to a feedback mechanism (38). Therefore, the signal might not be integrated via CtrA. However, the denitrification and flagellar gene clusters are also part of the Crp/Fnr superfamily regulon. They sense and react to oxygen and nitrogen species, e.g., to respond to the decreasing oxygen concentration at the end of an algal bloom (31, 44). Flagella, whose gene expression was inhibited by the sixth element, play an important role in the establishment of mutualistic symbioses with host algae (44, 57). However, while mutants of the Crp/Fnr superfamily led to inhibited CtrA regulon traits, the physiological shift from aerobic to anaerobic cultivation showed the contrary (44). Additional regulators of the CtrA regulon are either not regulated differently or downregulated in Dshi-6, like the partner-switching system of GafA and LexA (38, 47, 58, 59). Another activated protein in response to the 102-kb plasmid, which belongs to the CtrA regulon (60), is the cyclic di-GMP synthase diguanylate cyclase 2.

The genes controlled by the 102-kb plasmid are relevant under several environmental conditions, such as a switch from aerobic to anaerobic growth or the activation of the quorum sensing system (44). The greatest regulatory overlap was observed between the Dshi-6 and Δ86kb strains that antagonistically control a common regulon. In *D. shibae*, the QS synthase and regulator genes *luxI_3_* and *luxB* reside on the 86-kb plasmid. Both genes are controlled by CtrA, together with the chromosomally located *luxI_2_-luxR_2_* operon (58). The knockout of *luxI_2_* has an effect similar to that of the loss of the 86-kb plasmid (44). Thus, the part of the QS system acting downstream of CtrA might be involved in balancing the expression of denitrification and the CtrA regulon itself. A potential regulator found on the 102-kb plasmid is a sigma 70 factor. These sigma factors are components of the RNA polymerase and mediate the recognition of specific promoters, thus rapidly orchestrating complex cellular responses (61). Alternatively, the observed antagonistic gene expression could also result from copy number changes rather than regulators since the copy number of the 86-kb plasmid was reduced when the 102-kb plasmid was present.

Overall, the combination of the shared, antagonistic regulon and the copy number changes of the 86-kb plasmid in response to the presence of the 102-kb plasmid indicates a finely tuned regulation of traits, possibly connected to respiratory and cell communicative signals. Plasmid-chromosome cross-talks in *Alphaproteobacteria* have previously been reported, e.g., for the chromosomal gene expression of Methylorubrum extorquens AM1, which is controlled by the 44-kb plasmid pAM1-2 encoding transcription factors, small RNAs, and a QS synthase (62). In Sinorhizobium meliloti (63, 64), a megaplasmid regulates six genes on a chromid, which in turn differentially regulates 4% of the megaplasmid genes and 8% of the chromosomal genes (65, 66). Interestingly, this megaplasmid is being studied particularly in relation to its interaction with the host, but the possibility that it also controls chromosomal genes under certain conditions cannot be ruled out (67). An actual impact of a plasmid on the expression of 55 chromosomal genes was observed in Burkholderia cenocepacia (68).

### Plasmid loss, SNPs, and genetic bottlenecks: how representative are genetic mutants and type strains?

The current study exemplified for the 102-kb plasmid of *D. shibae* DFL 12^T^ that ECR losses might have severe effects on the physiological properties of a bacterium. Experimental curing of low-copy-number replicons is essentially based on plasmid incompatibility (32), but spontaneous losses, even of chromids, have been reported in *Roseobacteraceae*, e.g., for the 102-kb chromid pLA6_08 of Marinibacterium anthonyi DSM 107130 and the lifestyle-determining 262-kb antibiotic biosynthesis chromid of P. inhibens DSM 17395 (69). The absence of the 102-kb plasmid and an SNP in the ribosomal RpsA protein is indicative of a genetic bottleneck before the first deposit of the *D. shibae* DFL 12 type strain DSM 16493^T^ (Dshi-5). Such bottlenecks always occur when performing genetic knockouts and transposon library construction, etc., and in the future should be monitored by sequencing of the obtained mutants. Although the growth of *D. shibae* seemed not to be strongly affected by the loss of the 102-kb plasmid, the metabolic effects of an amino acid exchange in a ribosomal protein are unknown. Since the 102-kb plasmid has a large and probably even underestimated influence on chromosomal gene expression, a clonal lineage of strain Dshi-6 that is homogeneous with respect to the presence of the sixth element should be used in future experiments.

With respect to *D. shibae*, 16S rRNA gene sequences from more than two dozen strains, which were isolated mainly from micro- and macroalgae, have been deposited in the NCBI database (sequence identity of >99.4%). Complete genome sequencing of *D. shibae* DFL-36 (GenBank assembly accession number GCA_023558355.1), which was isolated from the phycosphere of the dinoflagellate Alexandrium ostenfeldii (69), revealed the presence of six ECRs that are nearly identical to those of *D. shibae* DFL 12 (Dshi-6) and lacked the characteristic SNP of Dshi-5. Our newly deposited *D. shibae* strain Dshi-6 (DFL 12 [DSM 112351]) is therefore much more representative of the species than Dshi-5.

### Outlook.

Cultivation and genetic manipulation carry the risks of selecting hidden SNPs, deletions, ECR losses, and large-scale chromosomal inversions (69). The bottleneck of a single bacterial cell affected by a spontaneous mutation can have serious physiological consequences for genetic engineering that are not caused by the actual manipulation. In the past, misleading interpretations were avoided by the establishment of independent mutants and genetic complementation. In the postgenomics era, whole-genome sequencing will be the method of choice to select defined mutants that ideally lack any further genetic modification. In addition, the isolation of new bacterial species initially represents a bottleneck situation. A final assessment of the genetic representativeness of a bacterial strain therefore depends on the availability of other isolates of the same species. These observations are relevant for biotechnologists and microbiologists working with mutants and new isolates.

## MATERIALS AND METHODS

### Bacterial strains.

All strains belong to the species *D. shibae*, and their genotypes are listed in Table 1.

**TABLE 1:** Dinoroseobacter shibae DFL 12^T^ strains used in this study^*a*^

Strain	Description	Reference
Dshi-5	DSM 16493^T^ with 5 ECRs	79
Δ72kb	DSM 16493^T^ with 4 ECRs lacking the 72-kb chromid	37
Δ86kb	DSM 16493^T^ with 4 ECRs lacking the 86-kb plasmid	13
Δ191kb	DSM 16493^T^ with 4 ECRs lacking the 191-kb plasmid	34
Dshi-6	DSM 112351 with 6 ECRs	This study
Dshi-6::Gm_102kb	DSM 112351 with a mariner transposon gentamycin tag on the 102-kb plasmid	This study

a Δ72kb, Dshi-5 Δ72kb; Δ86kb, Dshi-5 Δ86kb; Δ191kb, Dshi-5 Δ191kb.

### Growth curves.

Precultures were prepared by inoculation of 20 mL of defined saltwater medium (SWM) with 5 mM succinate and incubation overnight. The optical density at 600 nm was determined photometrically and adjusted to an OD of 0.01. Ten wells were filled per culture. Growth in MB medium and SWM with 5 mM succinate was determined via the optical density at 600 nm and monitored over a period of 36 h every 30 min at 30°C with the Bioscreen C system. Plots were processed using the Bioconductor package in R.

### Heavy metal resistance tests.

Heavy metal resistance tests of Dshi-5 and Dshi-6 were performed in defined SWM with 5 mM succinate. One hundred microliters of SWM was supplemented with final concentrations of CuCl, CoCl_2_, ZnCl_2_, and AgNO_3_ ranging between 0 and 0.2 mM. The growth of both *D. shibae* strains was monitored in 24-well plates for 96 h, using the Infinite 200 Pro microplate reader (Tecan Trading AG, Switzerland).

### PFGE analysis.

Pulsed-field gel electrophoresis (PFGE) analysis of extrachromosomal elements from Dshi-6 was performed as previously described (34).

### Stability assay.

A *D. shibae* mutant with a gentamicin resistance cassette on the 102-kb plasmid (Tm6060) was obtained from a transposon library as described previously by Ebert et al. (32). According to our protocol, which is used by default to determine the stability of plasmid constructs (34), the strain was incubated in either marine broth (MB) or SWM with gentamicin (40 μg/mL) for 4 days at 28°C before 1% of the culture was transferred to marine broth without antibiotics and incubated for a further 10 days. Dilutions of the liquid culture were plated onto MB plates and incubated for 4 days. Fifty single colonies were picked and individually resuspended in 20 μL of H_2_O, and 3 μL of the suspension was then spotted onto either 0.5× MB plates with gentamicin (test) or 1.0× MB plates without gentamicin (control). To exclude the effects of the 100-fold-diluted antibiotic on the maintenance of the plasmid, the stability assay in MB medium was repeated with the preculture first grown on 0.5× MB plates with gentamicin and using an inoculating loop of cells for the main culturing experiment for 7 days.

### Colony PCR.

The presence of the 102-kb plasmid in single colonies was determined via a new duplex PCR approach using the 191-kb plasmid as a positive control. Primers specific for the 102-kb plasmid (P2496 [5′-TCAACCAATCAAACGCGCC-3′] and P2497 [5′-AACAACGCATCGCCCAAAG-3′]) and the 191-kb plasmid (P430 [5′-TCTGGCTGCGTGGTGGCTTTC-3′] and P431 [5′-TGCGCTATAGTGCTCTCAACA-3′]) allowed the coamplification of both fragments with sizes of 518 bp and 730 bp, respectively. Cell breakup of single *D. shibae* colonies, which were picked with yellow pipetting tips and resuspended in 10 μL H_2_O, was achieved with a series of heating and cooling steps (5 min 30 s at 96°C, 1 min 30 s at 50°C, 1 min 30 s at 96°C, 1 min at 45°C, 1 min at 96°C, and 1 min at 40°C). The cell debris was centrifuged, and 1 μL of the supernatant was used for DNA coamplification with both primer pairs and the One *Taq* QuickLoad DNA polymerase (New England BioLabs) in a volume of 20 μL under the following cycling conditions: (i) 1 cycle of 3 min at 94°C; (ii) 30 cycles of 30 s at 94°C, 30 s at 55°C, and 45 s at 68°C; and (iii) 1 cycle of 10 min at 68°C.

### Phenotypic microarray.

*D. shibae* Dshi-5 and Dshi-6 strains were tested for their ability to utilize 95 different carbon sources using the Biolog PM01 microplate (34). Biolog experiments were performed with freshly grown *D. shibae* cells that were resuspended in 10 mL inoculating fluid IF-0 to achieve 85% transmittance in the Biolog turbidimeter and with a negative control without bacteria. A solution containing 428 μL H_2_O, 120 μL dye D, 1,200 μL 10× seawater medium (160 g NaCl, 32 g NaSO_4_, 24 g MgCl_2_ · 6H_2_O, 4 g KCl, 2 g NH_4_Cl, 1.6 g KH_2_PO_4_, and 1.2 g CaCl_2_ · 2H_2_O per L), 120 μL 100× NaHCO_3_ buffer (1.9 g/100 mL), 120 μL 100× vitamin mix (2 mg biotin [B_7_], 20 mg niacin [B_3_], 8 mg 4-aminobenzoic acid [B_10_], and 1 mg thiamine [B_1_] per L), and 12 μL 1,000× trace element solution (2.1 g FeSO_4_ · 7H_2_O, 13 mL 25% HCl, 5.2 g Na_2_EDTA, 30 mg H_3_BO_3_, 100 mg MnCl_2_ · 4H_2_O, 190 mg CoCl_2_ · 6H_2_O, 24 mg NiCl_2_ · 6H_2_O, 2 mg CuCl_2_ · 2H_2_O, 144 mg ZnSO_4_ · 7H_2_O, and 36 mg Na_2_MoO_4_ · 2H_2_O per liter) was added to a final volume of 12 mL. Each well of the PM01 plate was inoculated with the cell suspension, and the microplates were incubated at 28°C for 96 h in the OmniLog plate reader. The OmniLog data were processed and analyzed using the R package opm v1.1.0 (70). Curve parameters were estimated using spline fitting, and the curve maximum was used for plotting the heatmap and comparing the strains.

### Cultivation and sampling for transcriptome analysis.

Cultivation for transcriptome analysis was performed in three biological replicates. Precultures of all *D. shibae* strains were cultivated overnight at 28°C in MB medium. One hundred milliliters of MB medium without antibiotics was inoculated with 2 mL of the preculture, and the culture was grown in 250-mL Erlenmeyer flasks under agitation for about 9 h up to an OD_600_ of 0.6. Bacterial cells were centrifuged, and the pellets without remaining medium were frozen in liquid nitrogen, pestled, and stored in aliquots of about 150 mg at −80°C.

### RNA isolation and sequencing.

The pestled bacterial cell powder was covered with 1 mL TRIzol reagent (Thermo Fisher Scientific, Waltham, MA, USA) and incubated for 5 min at room temperature. Total RNA isolation starting with centrifugation at 12,000 × *g* for 10 min at 4°C was performed with the RNeasy minikit (Qiagen, Hilden, Germany) as previously described (57). After DNase treatment, the total RNA was resolved in 50 μL of diethyl pyrocarbonate (DEPC)-treated H_2_O. The depletion of rRNA was performed with the mRNA Ribo-Zero magnetic kit (Epicentre, Madison, WI, USA) according to the manufacturer’s instructions. The library was prepared from ribosomally depleted total RNA using the Scriptseq v2 RNA-seq (RNA sequencing) library preparation kit (Epicentre, Madison, WI, USA) according to the manufacturer’s protocol. For sequencing, equal volumes of libraries (12 pM) were multiplexed on a single lane. Cluster generation was performed with cBot (Illumina, San Diego, CA, USA) using TruSeq SR cluster kit v3-cBot-HS (Illumina, San Diego, CA, USA). Sequencing was done on the HiSeq 2500 platform (Illumina, San Diego, CA, USA) using TruSeq SBS kit v3-HS (Illumina, San Diego, CA, USA) for 50 cycles. Image analysis and base calling were performed using the Illumina pipeline v1.8 (Illumina, San Diego, CA, USA).

### RNA-seq data processing and analysis.

The demultiplexed raw fastq files were quality controlled using the FASTQ-mcf suite (https://github.com/ExpressionAnalysis/ea-utils). Low-quality bases and identified Illumina adaptors were clipped from the sequences. Reads were mapped to reference genomes using bowtie2 with default parameters (71). Ambiguously mapping reads were equally distributed among all regions to which they could be assigned. The resulting sam files were converted to binary format and indexed using samtools (72). A table with reads per protein-coding sequence was generated from each indexed bam file using featureCounts (72). The R package EdgeR was used for statistical analysis of differential gene expression (72). All strains were compared with the *D. shibae* reference strain DSM 16493^T^. Data for all genes can be found in Table S1A. The R package KEGGREST v1.1 (73) was used to extract the KEGG (74) annotation for *D. shibae* (accessed 1 April 2020). A hypergeometric test was used to identify significantly enriched KEGG categories.

### Genome sequencing.

Genomic DNA from all *D. shibae* strains was isolated with the Qiagen (Hilden, Germany) genomic DNA kit. Illumina and PacBio library preparation, sequencing, PacBio genome assembly, and error correction with Illumina reads were conducted as previously described (48, 75).

### Comparative genomics.

The sequence alignment of the 102-kb plasmid with other sequences was done using mauve v20150226 (76, 77). Further processing was done in R using the genoPlotR package v0.8.11 (http://genoplotr.r-forge.r-project.org/). Homologous genes among the 102-kb plasmid, the Phaeobacter inhibens plasmid Pp88, and Roseobacter litoralis Och 149 or the extrachromosomal replicons of *D. shibae* were identified using ProteinOrtho (78) by applying the following settings: E value of 1e−05, identity of 30, and coverage of 75.

The dendrogram for the differentiation of chromids and plasmids is based on relative synonymous codon usage analysis of all protein-coding genes of the replicons (23).

### Data availability.

RNA sequencing data, which were established in three biological replicates, have been deposited at the NCBI Gene Expression Omnibus database under accession number GSE198318.

## References

[1] Wein T, Dagan T. 2020. Plasmid evolution. Curr Biol 30:R1158–R1163. doi:10.1016/j.cub.2020.07.003. 33022260

[2] Mathers AJ, Peirano G, Pitout JDD. 2015. The role of epidemic resistance plasmids and international high-risk clones in the spread of multidrug-resistant Enterobacteriaceae. Clin Microbiol Rev 28:565–591. doi:10.1128/CMR.00116-14. 25926236 PMC4405625

[3] Brinkmann H, Göker M, Koblížek M, Wagner-Döbler I, Petersen J. 2018. Horizontal operon transfer, plasmids, and the evolution of photosynthesis in Rhodobacteraceae. ISME J 12:1994–2010. doi:10.1038/s41396-018-0150-9. 29795276 PMC6052148

[4] Rankin DJ, Rocha EPC, Brown SP. 2011. What traits are carried on mobile genetic elements, and why. Heredity (Edinb) 106:1–10. doi:10.1038/hdy.2010.24. 20332804 PMC3183850

[5] Dragoš A, Kiesewalter H, Martin M, Hsu C-Y, Hartmann R, Wechsler T, Eriksen C, Brix S, Drescher K, Stanley-Wall N, Kümmerli R, Kovács ÁT. 2018. Division of labor during biofilm matrix production. Curr Biol 28:1903–1913.e5. doi:10.1016/j.cub.2018.04.046. 29887307 PMC6331042

[6] Sheppard AE, Stoesser N, Wilson DJ, Sebra R, Kasarskis A, Anson LW, Giess A, Pankhurst LJ, Vaughan A, Grim CJ, Cox HL, Yeh AJ, Modernising Medical Microbiology (MMM) Informatics Group, Sifri CD, Walker AS, Peto TE, Crook DW, Mathers AJ. 2016. Nested Russian doll-like genetic mobility drives rapid dissemination of the carbapenem resistance gene bla_KPC_. Antimicrob Agents Chemother 60:3767–3778. doi:10.1128/AAC.00464-16. 27067320 PMC4879409

[7] Hall JPJ, Williams D, Paterson S, Harrison E, Brockhurst MA. 2017. Positive selection inhibits gene mobilization and transfer in soil bacterial communities. Nat Ecol Evol 1:1348–1353. doi:10.1038/s41559-017-0250-3. 28890938 PMC5584672

[8] Sun L, Alexander HK, Bogos B, Kiviet DJ, Ackermann M, Bonhoeffer S. 2018. Effective polyploidy causes phenotypic delay and influences bacterial evolvability. PLoS Biol 16:e2004644. doi:10.1371/journal.pbio.2004644. 29470493 PMC5839593

[9] Rodríguez-Beltrán J, Sørum V, Toll-Riera M, de la Vega C, Peña-Miller R, Millán ÁS. 2020. Genetic dominance governs the evolution and spread of mobile genetic elements in bacteria. Proc Natl Acad Sci USA 117:15755–15762. doi:10.1073/pnas.2001240117. 32571917 PMC7355013

[10] Barton IS, Eagan JL, Nieves-Otero PA, Reynolds IP, Platt TG, Fuqua C. 2021. Co-dependent and interdigitated: dual quorum sensing systems regulate conjugative transfer of the Ti plasmid and the At megaplasmid in Agrobacterium tumefaciens 15955. Front Microbiol 11:605896. doi:10.3389/fmicb.2020.605896. 33552018 PMC7856919

[11] Baharoglu Z, Bikard D, Mazel D. 2010. Conjugative DNA transfer induces the bacterial SOS response and promotes antibiotic resistance development through integron activation. PLoS Genet 6:e1001165. doi:10.1371/journal.pgen.1001165. 20975940 PMC2958807

[12] Rodríguez-Beltrán J, DelaFuente J, León-Sampedro R, MacLean RC, San Millán Á. 2021. Beyond horizontal gene transfer: the role of plasmids in bacterial evolution. Nat Rev Microbiol 19:347–359. doi:10.1038/s41579-020-00497-1. 33469168

[13] Maslowska KH, Makiela-Dzbenska K, Fijalkowska IJ. 2019. The SOS system: a complex and tightly regulated response to DNA damage. Environ Mol Mutagen 60:368–384. doi:10.1002/em.22267. 30447030 PMC6590174

[14] Kyslik P, Dobisova M, Maresova H, Sobotkova L. 1993. Plasmid burden in chemostat culture of Escherichia coli: its effect on the selection for overproducers of host enzymes. Biotechnol Bioeng 41:325–329. doi:10.1002/bit.260410306. 18609556

[15] Wong Ng J, Chatenay D, Robert J, Poirier MG. 2010. Plasmid copy number noise in monoclonal populations of bacteria. Phys Rev E Stat Nonlin Soft Matter Phys 81:011909. doi:10.1103/PhysRevE.81.011909. 20365401

[16] Trautwein K, Will SE, Hulsch R, Maschmann U, Wiegmann K, Hensler M, Michael V, Ruppersberg H, Wünsch D, Feenders C, Neumann-Schaal M, Kaltenhäuser S, Ulbrich M, Schmidt-Hohagen K, Blasius B, Petersen J, Schomburg D, Rabus R. 2016. Native plasmids restrict growth of Phaeobacter inhibens DSM 17395: energetic costs of plasmids assessed by quantitative physiological analyses. Environ Microbiol 18:4817–4829. doi:10.1111/1462-2920.13381. 27233797

[17] Harms A, Brodersen DE, Mitarai N, Gerdes K. 2018. Toxins, targets, and triggers: an overview of toxin-antitoxin biology. Mol Cell 70:768–784. doi:10.1016/j.molcel.2018.01.003. 29398446

[18] Slater FR, Bailey MJ, Tett AJ, Turner SL. 2008. Progress towards understanding the fate of plasmids in bacterial communities. FEMS Microbiol Ecol 66:3–13. doi:10.1111/j.1574-6941.2008.00505.x. 18507680

[19] Harold FM. 1986. The vital force: a study of bioenergetics. WH Freeman & Co, New York, NY.

[20] Liang KYH, Orata FD, Boucher YF, Case RJ. 2021. Roseobacters in a sea of poly- and paraphyly: whole genome-based taxonomy of the family Rhodobacteraceae and the proposal for the split of the “Roseobacter clade” into a novel family, Roseobacteraceae fam. nov. Front Microbiol 12:683109. doi:10.3389/fmicb.2021.683109. 34248901 PMC8267831

[21] Luo H, Moran MA. 2014. Evolutionary ecology of the marine Roseobacter clade. Microbiol Mol Biol Rev 78:573–587. doi:10.1128/MMBR.00020-14. 25428935 PMC4248658

[22] Wagner-Döbler I, Biebl H. 2006. Environmental biology of the marine Roseobacter lineage. Annu Rev Microbiol 60:255–280. doi:10.1146/annurev.micro.60.080805.142115. 16719716

[23] Petersen J, Frank O, Göker M, Pradella S. 2013. Extrachromosomal, extraordinary and essential—the plasmids of the Roseobacter clade. Appl Microbiol Biotechnol 97:2805–2815. doi:10.1007/s00253-013-4746-8. 23435940

[24] Petersen J, Brinkmann H, Pradella S. 2009. Diversity and evolution of repABC type plasmids in Rhodobacterales. Environ Microbiol 11:2627–2638. doi:10.1111/j.1462-2920.2009.01987.x. 19601964

[25] Michael V, Frank O, Bartling P, Scheuner C, Göker M, Brinkmann H, Petersen J. 2016. Biofilm plasmids with a rhamnose operon are widely distributed determinants of the ‘swim-or-stick’ lifestyle in roseobacters. ISME J 10:2498–2513. doi:10.1038/ismej.2016.30. 26953602 PMC5030684

[26] Frank O, Michael V, Päuker O, Boedeker C, Jogler C, Rohde M, Petersen J. 2015. Plasmid curing and the loss of grip—the 65-kb replicon of Phaeobacter inhibens DSM 17395 is required for biofilm formation, motility and the colonization of marine algae. Syst Appl Microbiol 38:120–127. doi:10.1016/j.syapm.2014.12.001. 25595869

[27] Brinkhoff T, Bach G, Heidorn T, Liang L, Schlingloff A, Simon M. 2004. Antibiotic production by a Roseobacter clade-affiliated species from the German Wadden Sea and its antagonistic effects on indigenous isolates. Appl Environ Microbiol 70:2560–2565. doi:10.1128/AEM.70.4.2560-2565.2003. 15066861 PMC383154

[28] Wagner-Döbler I, Ballhausen B, Berger M, Brinkhoff T, Buchholz I, Bunk B, Cypionka H, Daniel R, Drepper T, Gerdts G, Hahnke S, Han C, Jahn D, Kalhoefer D, Kiss H, Klenk H-P, Kyrpides N, Liebl W, Liesegang H, Meincke L, Pati A, Petersen J, Piekarski T, Pommerenke C, Pradella S, Pukall R, Rabus R, Stackebrandt E, Thole S, Thompson L, Tielen P, Tomasch J, von Jan M, Wanphrut N, Wichels A, Zech H, Simon M. 2010. The complete genome sequence of the algal symbiont Dinoroseobacter shibae: a hitchhiker’s guide to life in the sea. ISME J 4:61–77. doi:10.1038/ismej.2009.94. 19741735

[29] Patzelt D, Wang H, Buchholz I, Rohde M, Gröbe L, Pradella S, Neumann A, Schulz S, Heyber S, Münch K, Münch R, Jahn D, Wagner-Döbler I, Tomasch J. 2013. You are what you talk: quorum sensing induces individual morphologies and cell division modes in Dinoroseobacter shibae. ISME J 7:2274–2286. doi:10.1038/ismej.2013.107. 23823498 PMC3834844

[30] Tomasch J, Wang H, Hall ATK, Patzelt D, Preusse M, Petersen J, Brinkmann H, Bunk B, Bhuju S, Jarek M, Geffers R, Lang AS, Wagner-Döbler I. 2018. Packaging of Dinoroseobacter shibae DNA into gene transfer agent particles is not random. Genome Biol Evol 10:359–369. doi:10.1093/gbe/evy005. 29325123 PMC5786225

[31] Ebert M, Laaß S, Thürmer A, Roselius L, Eckweiler D, Daniel R, Härtig E, Jahn D. 2017. FnrL and three Dnr regulators are used for the metabolic adaptation to low oxygen tension in Dinoroseobacter shibae. Front Microbiol 8:642. doi:10.3389/fmicb.2017.00642. 28473807 PMC5398030

[32] Ebert M, Laaß S, Burghartz M, Petersen J, Koßmehl S, Wöhlbrand L, Rabus R, Wittmann C, Tielen P, Jahn D. 2013. Transposon mutagenesis identified chromosomal and plasmid genes essential for adaptation of the marine bacterium Dinoroseobacter shibae to anaerobic conditions. J Bacteriol 195:4769–4777. doi:10.1128/JB.00860-13. 23974024 PMC3807425

[33] Patzelt D, Michael V, Päuker O, Ebert M, Tielen P, Jahn D, Tomasch J, Petersen J, Wagner-Döbler I. 2016. Gene flow across genus barriers—conjugation of Dinoroseobacter shibae’s 191-kb killer plasmid into Phaeobacter inhibens and AHL-mediated expression of type IV secretion systems. Front Microbiol 7:742. doi:10.3389/fmicb.2016.00742. 27303368 PMC4886583

[34] Wang H, Tomasch J, Michael V, Bhuju S, Jarek M, Petersen J, Wagner-Döbler I. 2015. Identification of genetic modules mediating the Jekyll and Hyde interaction of Dinoroseobacter shibae with the dinoflagellate Prorocentrum minimum. Front Microbiol 6:1262. doi:10.3389/fmicb.2015.01262. 26617596 PMC4643747

[35] Mansky J, Wang H, Ebert M, Härtig E, Jahn D, Tomasch J, Wagner-Döbler I. 2022. The influence of genes on the “killer plasmid” of Dinoroseobacter shibae on its symbiosis with the dinoflagellate Prorocentrum minimum. Front Microbiol 12:804767. doi:10.3389/fmicb.2021.804767. 35154034 PMC8831719

[36] Kleist S, Ulbrich M, Bill N, Schmidt-Hohagen K, Geffers R, Schomburg D. 2017. Dealing with salinity extremes and nitrogen limitation—an unexpected strategy of the marine bacterium Dinoroseobacter shibae. Environ Microbiol 19:894–908. doi:10.1111/1462-2920.13266. 26914854

[37] Soora M, Tomasch J, Wang H, Michael V, Petersen J, Engelen B, Wagner-Döbler I, Cypionka H. 2015. Oxidative stress and starvation in Dinoroseobacter shibae: the role of extrachromosomal elements. Front Microbiol 6:233. doi:10.3389/fmicb.2015.00233. 25859246 PMC4373377

[38] Koppenhöfer S, Wang H, Scharfe M, Kaever V, Wagner-Döbler I, Tomasch J. 2019. Integrated transcriptional regulatory network of quorum sensing, replication control, and SOS response in Dinoroseobacter shibae. Front Microbiol 10:803. doi:10.3389/fmicb.2019.00803. 31031742 PMC6473078

[39] Pradella S, Allgaier M, Hoch C, Päuker O, Stackebrandt E, Wagner-Döbler I. 2004. Genome organization and localization of the pufLM genes of the photosynthesis reaction center in phylogenetically diverse marine Alphaproteobacteria. Appl Environ Microbiol 70:3360–3369. doi:10.1128/AEM.70.6.3360-3369.2004. 15184132 PMC427745

[40] Allgaier M, Uphoff H, Felske A, Wagner-Döbler I. 2003. Aerobic anoxygenic photosynthesis in Roseobacter clade bacteria from diverse marine habitats. Appl Environ Microbiol 69:5051–5059. doi:10.1128/AEM.69.9.5051-5059.2003. 12957886 PMC194994

[41] Tomasch J, Ringel V, Wang H, Freese HM, Bartling P, Brinkmann H, Vollmers J, Jarek M, Wagner-Döbler I, Petersen J. 2022. Fatal affairs—conjugational transfer of a dinoflagellate-killing plasmid between marine Rhodobacterales. Microb Genom 8:000787. doi:10.1099/mgen.0.000787. PMC9176285 35254236

[42] Petersen J, Brinkmann H, Berger M, Brinkhoff T, Päuker O, Pradella S. 2011. Origin and evolution of a novel DnaA-like plasmid replication type in Rhodobacterales. Mol Biol Evol 28:1229–1240. doi:10.1093/molbev/msq310. 21097494

[43] Berland BR, Bonin DJ, Maestrini SY. 1969. Study of bacteria associated with marine algae in culture. Mar Biol 3:334–335. doi:10.1007/BF00698862.

[44] Koppenhöfer S, Lang AS. 2020. Interactions among redox regulators and the CtrA phosphorelay in Dinoroseobacter shibae and Rhodobacter capsulatus. Microorganisms 8:562. doi:10.3390/microorganisms8040562. PMC7232146 32295208

[45] Laub MT, Chen SL, Shapiro L, Mcadams HH. 2002. Genes directly controlled by CtrA, a master regulator of the Caulobacter cell cycle. Proc Natl Acad Sci USA 99:4632–4637. doi:10.1073/pnas.062065699. 11930012 PMC123699

[46] Mignolet J, Panis GL, Viollier PH. 2018. More than a Tad: spatiotemporal control of Caulobacter pili. Curr Opin Microbiol 42:79–86. doi:10.1016/j.mib.2017.10.017. 29161615

[47] Fogg PCM. 2019. Identification and characterization of a direct activator of a gene transfer agent. Nat Commun 10:595. doi:10.1038/s41467-019-08526-1. 30723210 PMC6363796

[48] Bartling P, Brinkmann H, Bunk B, Overmann J, Göker M, Petersen J. 2017. The composite 259-kb plasmid of Martelella mediterranea DSM 17316T—a natural replicon with functional RepABC modules from Rhodobacteraceae and Rhizobiaceae. Front Microbiol 8:1787. doi:10.3389/fmicb.2017.01787. 28983283 PMC5613091

[49] Carroll AC, Wong A. 2018. Plasmid persistence: costs, benefits, and the plasmid paradox. Can J Microbiol 64:293–304. doi:10.1139/cjm-2017-0609. 29562144

[50] San Millan A, MacLean RC. 2017. Fitness costs of plasmids: a limit to plasmid transmission. Microbiol Spectr 5:MTBP-0016-2017. doi:10.1128/microbiolspec.MTBP-0016-2017. PMC11687550 28944751

[51] Will SE, Neumann-Schaal M, Heydorn RL, Bartling P, Petersen J, Schomburg D. 2017. The limits to growth—energetic burden of the endogenous antibiotic tropodithietic acid in Phaeobacter inhibens DSM 17395. PLoS One 12:e0177295. doi:10.1371/journal.pone.0177295. 28481933 PMC5421792

[52] Gordon JE, Christie PJ. 2014. The Agrobacterium Ti plasmids. Microbiol Spectr 2:PLAS-0010-2013. doi:10.1128/microbiolspec.PLAS-0010-2013. PMC4292801 25593788

[53] Cevallos MA, Cervantes-Rivera R, Gutiérrez-Ríos RM. 2008. The repABC plasmid family. Plasmid 60:19–37. doi:10.1016/j.plasmid.2008.03.001. 18433868

[54] Pinto UM, Pappas KM, Winans SC. 2012. The ABCs of plasmid replication and segregation. Nat Rev Microbiol 10:755–765. doi:10.1038/nrmicro2882. 23070556

[55] González V, Santamaría RI, Bustos P, Hernández-González I, Medrano-Soto A, Moreno-Hagelsieb G, Janga SC, Ramírez MA, Jiménez-Jacinto V, Collado-Vides J, Dávila G. 2006. The partitioned Rhizobium etli genome: genetic and metabolic redundancy in seven interacting replicons. Proc Natl Acad Sci USA 103:3834–3839. doi:10.1073/pnas.0508502103. 16505379 PMC1383491

[56] Raymond C, Tom R, Perret S, Moussouami P, L’Abbé D, St-Laurent G, Durocher Y. 2011. A simplified polyethylenimine-mediated transfection process for large-scale and high-throughput applications. Methods 55:44–51. doi:10.1016/j.ymeth.2011.04.002. 21539918

[57] Wang H, Tomasch J, Jarek M, Wagner-Döbler I. 2014. A dual-species co-cultivation system to study the interactions between roseobacters and dinoflagellates. Front Microbiol 5:311. doi:10.3389/fmicb.2014.00311. 25009539 PMC4069834

[58] Wang H, Ziesche L, Frank O, Michael V, Martin M, Petersen J, Schulz S, Wagner-Döbler I, Tomasch J. 2014. The CtrA phosphorelay integrates differentiation and communication in the marine alphaproteobacterium Dinoroseobacter shibae. BMC Genomics 15:130. doi:10.1186/1471-2164-15-130. 24524855 PMC4046655

[59] Westbye AB, Beatty JT, Lang AS, Rice P. 2017. Guaranteeing a captive audience: coordinated regulation of gene transfer agent (GTA) production and recipient capability by cellular regulators. Curr Opin Microbiol 38:122–129. doi:10.1016/j.mib.2017.05.003. 28599143

[60] Bedrunka P, Olbrisch F, Rüger M, Zehner S, Frankenberg-Dinkel N. 2018. Nitric oxide controls c-di-GMP turnover in Dinoroseobacter shibae. Microbiology (Reading) 164:1405–1415. doi:10.1099/mic.0.000714. 30222100

[61] Paget M, Helmann J. 2003. Protein family review—the sigma(70) family of sigma factors. Genome Biol 4:203–206. doi:10.1186/gb-2003-4-1-203. 12540296 PMC151288

[62] Nieto Penalver CG, Cantet F, Morin D, Haras D, Vorholt JA. 2006. A plasmid-borne truncated luxI homolog controls quorum-sensing systems and extracellular carbohydrate production in Methylobacterium extorquens AM1. J Bacteriol 188:7321–7324. doi:10.1128/JB.00649-06. 17015673 PMC1636247

[63] Hall JPJ, Wright RCT, Guymer D, Harrison E, Brockhurst MA. 2020. Extremely fast amelioration of plasmid fitness costs by multiple functionally diverse pathways. Microbiology (Reading) 166:56–62. doi:10.1099/mic.0.000862. 31613206

[64] Galardini M, Brilli M, Spini G, Rossi M, Roncaglia B, Bani A, Chiancianesi M, Moretto M, Engelen K, Bacci G, Pini F, Biondi EG, Bazzicalupo M, Mengoni A. 2015. Evolution of intra-specific regulatory networks in a multipartite bacterial genome. PLoS Comput Biol 11:e1004478. doi:10.1371/journal.pcbi.1004478. 26340565 PMC4560400

[65] Fei F, diCenzo GC, Bowdish DME, McCarry BE, Finan TM. 2016. Effects of synthetic large-scale genome reduction on metabolism and metabolic preferences in a nutritionally complex environment. Metabolomics 12:23. doi:10.1007/s11306-015-0928-y.

[66] diCenzo GC, Wellappili D, Brian Golding G, Finan TM. 2018. Inter-replicon gene flow contributes to transcriptional integration in the Sinorhizobium meliloti multipartite genome. G3 (Bethesda) 8:1711–1720. doi:10.1534/g3.117.300405. 29563186 PMC5940162

[67] Vial L, Hommais F. 2020. Plasmid‐chromosome cross‐talks. Environ Microbiol 22:540–556. doi:10.1111/1462-2920.14880. 31782608

[68] Agnoli K, Schwager S, Uehlinger S, Vergunst A, Viteri DF, Nguyen DT, Sokol PA, Carlier A, Eberl L. 2012. Exposing the third chromosome of Burkholderia cepacia complex strains as a virulence plasmid. Mol Microbiol 83:362–378. doi:10.1111/j.1365-2958.2011.07937.x. 22171913

[69] Wünsch D, Strijkstra A, Wöhlbrand L, Freese HM, Scheve S, Hinrichs C, Trautwein K, Maczka M, Petersen J, Schulz S, Overmann J, Rabus R. 2020. Global response of Phaeobacter inhibens DSM 17395 to deletion of its 262-kb chromid encoding antibiotic synthesis. Microb Physiol 30:9–24. doi:10.1159/000508591. 32958725

[70] Ding H, Moksa MM, Hirst M, Beatty JT. 2014. Draft genome sequences of six Rhodobacter capsulatus strains, YW1, YW2, B6, Y262, R121, and DE442. Genome Announc 2:e00050-14. doi:10.1128/genomeA.00050-14. 24526637 PMC3924369

[71] Langmead B, Salzberg SL. 2012. Fast gapped-read alignment with Bowtie 2. Nat Methods 9:357–359. doi:10.1038/nmeth.1923. 22388286 PMC3322381

[72] Li H, Handsaker B, Wysoker A, Fennell T, Ruan J, Homer N, Marth G, Abecasis G, Durbin R, 1000 Genome Project Data Processing Subgroup. 2009. The Sequence Alignment/Map format and SAMtools. Bioinformatics 25:2078–2079. doi:10.1093/bioinformatics/btp352. 19505943 PMC2723002

[73] Tenenbaum D. 2016. KEGGREST: client-side REST access to KEGG.

[74] Kanehisa M, Goto S. 2000. KEGG: Kyoto Encyclopedia of Genes and Genomes. Nucleic Acids Res 28:27–30. doi:10.1093/nar/28.1.27. 10592173 PMC102409

[75] Yoon MY, Lee K-M, Park Y, Yoon SS. 2011. Contribution of cell elongation to the biofilm formation of Pseudomonas aeruginosa during anaerobic respiration. PLoS One 6:e16105. doi:10.1371/journal.pone.0016105. 21267455 PMC3022656

[76] Darling ACE, Mau B, Blattner FR, Perna NT. 2004. Mauve: multiple alignment of conserved genomic sequence with rearrangements. Genome Res 14:1394–1403. doi:10.1101/gr.2289704. 15231754 PMC442156

[77] Darling AE, Mau B, Perna NT. 2010. Progressivemauve: multiple genome alignment with gene gain, loss and rearrangement. PLoS One 5:e11147. doi:10.1371/journal.pone.0011147. 20593022 PMC2892488

[78] Lechner M, Findeiss S, Steiner L, Marz M, Stadler PF, Prohaska SJ. 2011. Proteinortho: detection of (co-)orthologs in large-scale analysis. BMC Bioinformatics 12:124. doi:10.1186/1471-2105-12-124. 21526987 PMC3114741

[79] Biebl H, Allgaier M, Tindall BJ, Koblizek M, Lünsdorf H, Pukall R, Wagner-Döbler I. 2005. Dinoroseobacter shibae gen. nov., sp. nov., a new aerobic phototrophic bacterium isolated from dinoflagellates. Int J Syst Evol Microbiol 55:1089–1096. doi:10.1099/ijs.0.63511-0. 15879238

