# Supplementary material for: The Sixth Element: a 102-kb RepABC Plasmid of Xenologous Origin Modulates Chromosomal Gene Expression in Dinoroseobacter shibae
Source: mSystems. 2022 Aug 3;7(4):e00264-22. doi: 10.1128/msystems.00264-22 (PMC9426580; doi:10.1128/msystems.00264-22)
Supplement: FIG S1 [file msystems.00264-22-s0001.docx]

**Supplementary figures**


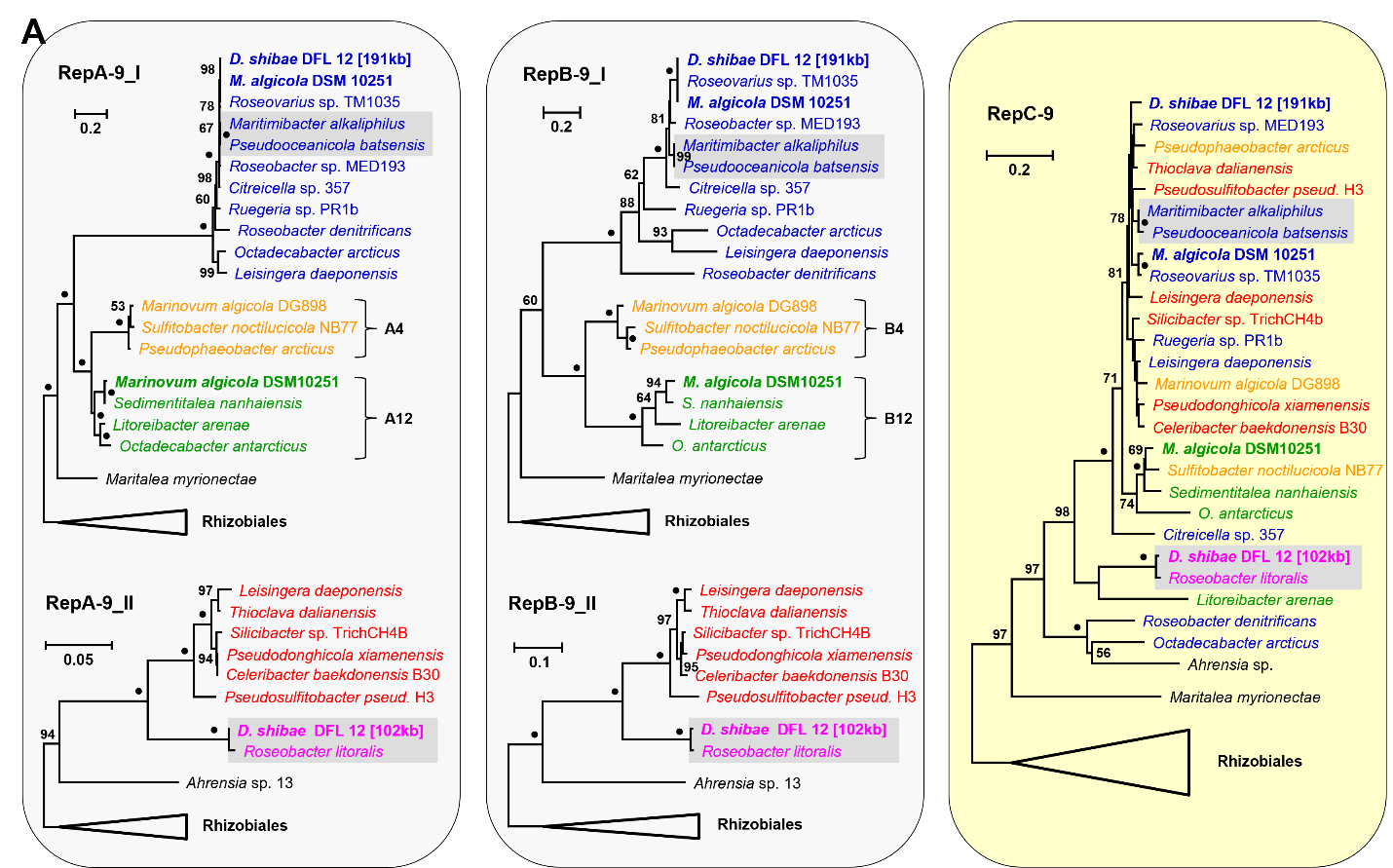

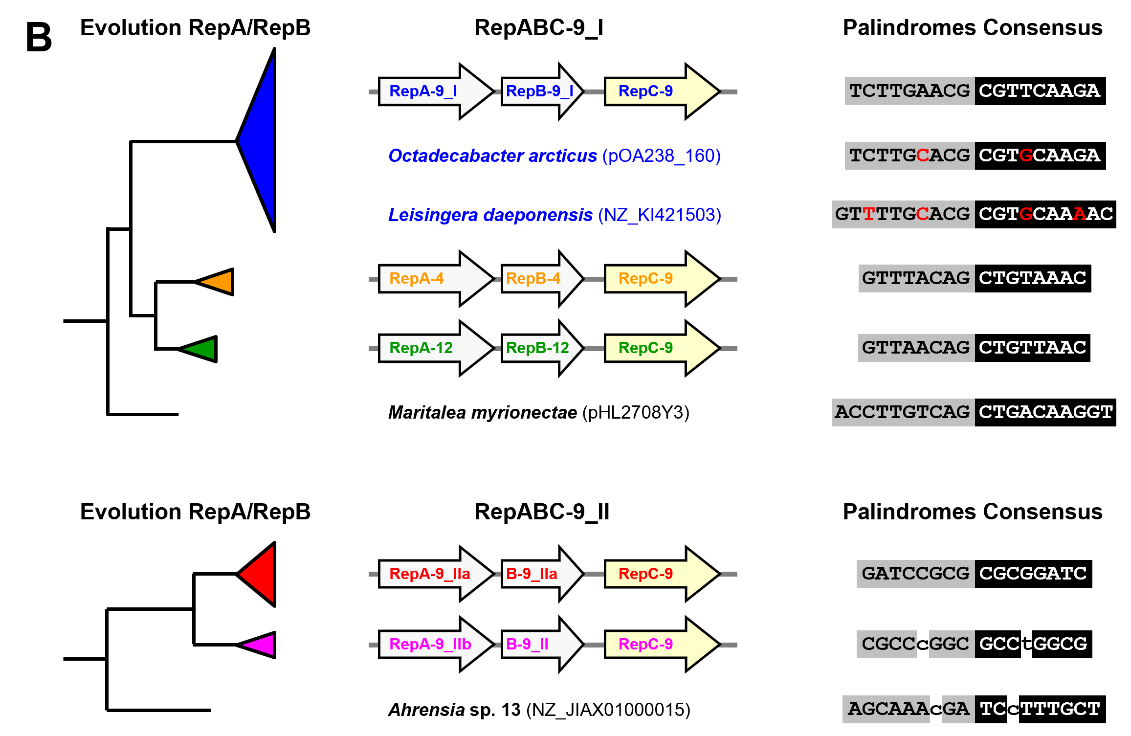


Figure S1: **A**) Phylogenetic protein analyses of RepABC-9 type plasmid replication modules of Rhodobacterales. The complete set of RepC-9 replicase sequences and the adjacent RepA and RepB partitioning genes have been analyzed with rhizobial outgroup sequences (supplementary Table S3). Color code indicates putative compatibility groups of RepABC-9 type replication modules: red, RepABC-9_I; orange, A4-B4-C9; green, A12-B12-C9; red, RepABC-9_IIa; orange, RepABC-9_IIb. RepA, RepB and RepC-9 phylogenies are presented with a bright gray and a yellow background, respectively. Taxa with two RepABC-9 type plasmids are shown in bold. Dark gray boxes highlight a synchronous evolution of RepA, RepB and RepC-9 proteins. ●, 100% bootstrap support. **B)** Consensus palindrome sequences of RepABC-9 type plasmids according to the phylogenetic analyses (Figure S1A).
