# Supplementary material for: The Sixth Element: a 102-kb RepABC Plasmid of Xenologous Origin Modulates Chromosomal Gene Expression in Dinoroseobacter shibae
Source: mSystems. 2022 Aug 3;7(4):e00264-22. doi: 10.1128/msystems.00264-22 (PMC9426580; doi:10.1128/msystems.00264-22)
Supplement: FIG S2 [file msystems.00264-22-s0002.docx]

Figure S2: Comparison of the gene expression of all seven replicons from Dshi-6. The box plots of the three biological replicates (rep1, rep2, rep3) are shown in a logarithmic scale. Median expression values of the replicons from replicate 1 are the following; chromosome: 2206x (NC_009952.1), pDSHI01: 1713x (NC_009955.1), pDSHI02: 1228x (NC_009956.1), pDSHI03: 1740x (NC_009957.1), pDSHI04: 2529x (NC_009958.1), pDSHI05: 1008x (NC_009959.1), pDSHI06: 501x (CP097546.1).
