# Supplementary material for: The Sixth Element: a 102-kb RepABC Plasmid of Xenologous Origin Modulates Chromosomal Gene Expression in Dinoroseobacter shibae
Source: mSystems. 2022 Aug 3;7(4):e00264-22. doi: 10.1128/msystems.00264-22 (PMC9426580; doi:10.1128/msystems.00264-22)
Supplement: FIG S3 [file msystems.00264-22-s0003.docx]

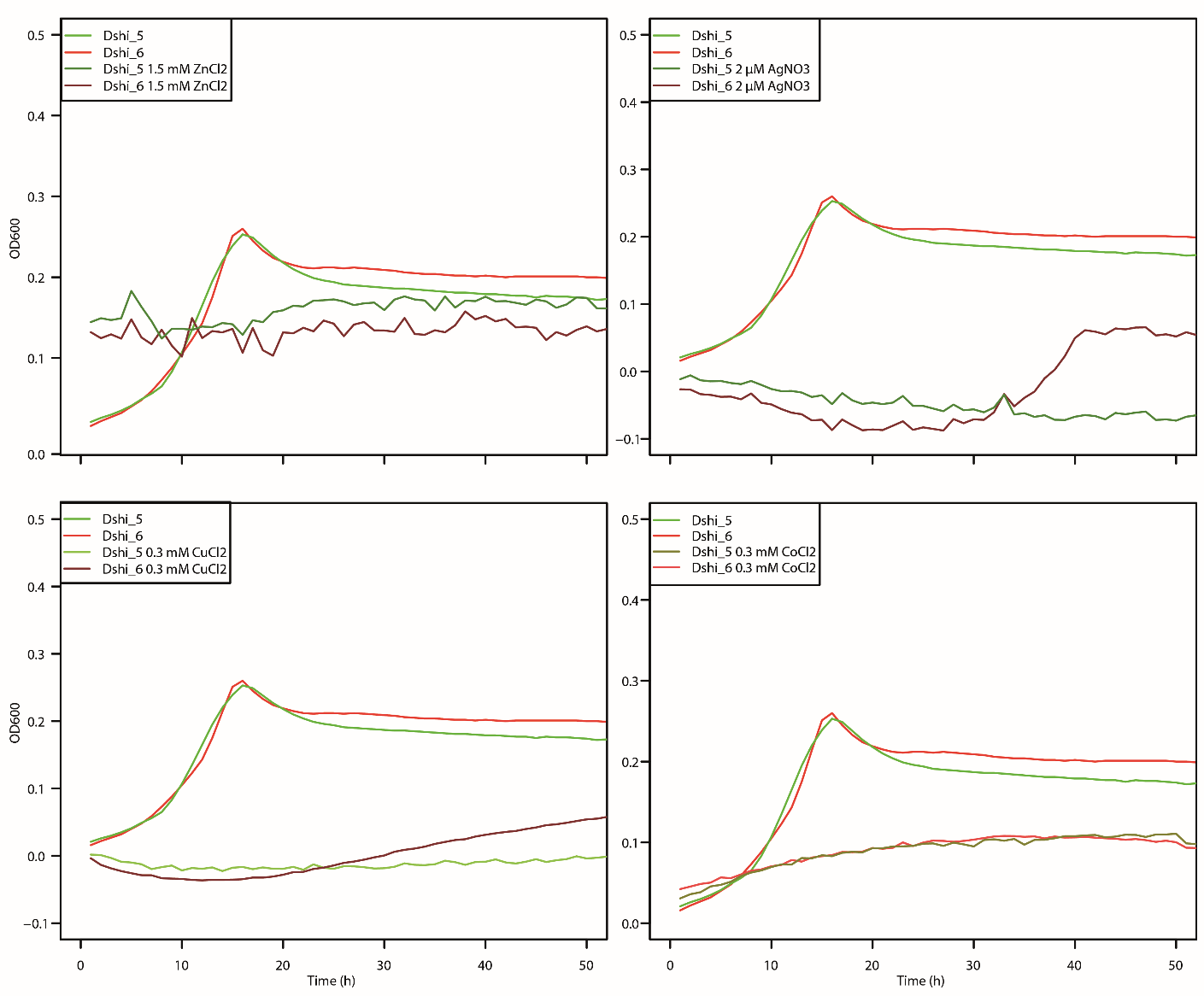


Figure S3: Growth kinetics of strains Dshi-5 (five ECRs) and Dshi-6 (six ECRs) in ASW medium with succinate and different heavy metals. Growth was monitored over 50-70h and measured at an optical density of 600 nm.
