# Supplementary material for: The Sixth Element: a 102-kb RepABC Plasmid of Xenologous Origin Modulates Chromosomal Gene Expression in Dinoroseobacter shibae
Source: mSystems. 2022 Aug 3;7(4):e00264-22. doi: 10.1128/msystems.00264-22 (PMC9426580; doi:10.1128/msystems.00264-22)
Supplement: FIG S4 [file msystems.00264-22-s0004.docx]

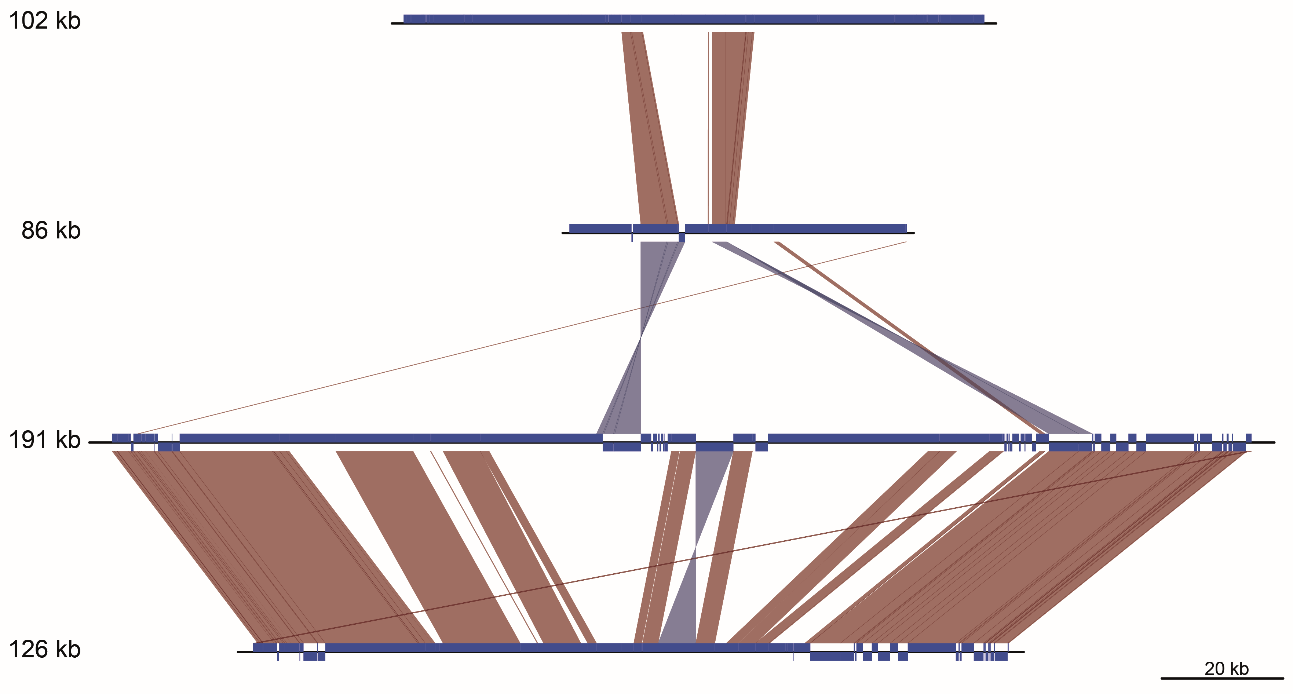


Figure S4A: Sequence alignment of the 102 kb plasmid with three other D. shibae replicons. The Mauve alignment tool and R package genoPlotR were used for generation of the alignment and visualization. The homologous areas between the 102 kb plasmid and the 86 kb and 126 kb plasmids are the genes Dshi_4092 and DSHI_RS22065 or DSHI_RS22030 and Dshi_4035, which are two transposases each. In the plasmid 191 kb, the genes Dshi_3701 and Dshi_3700 show a homology, these are an integrase and a transposase.


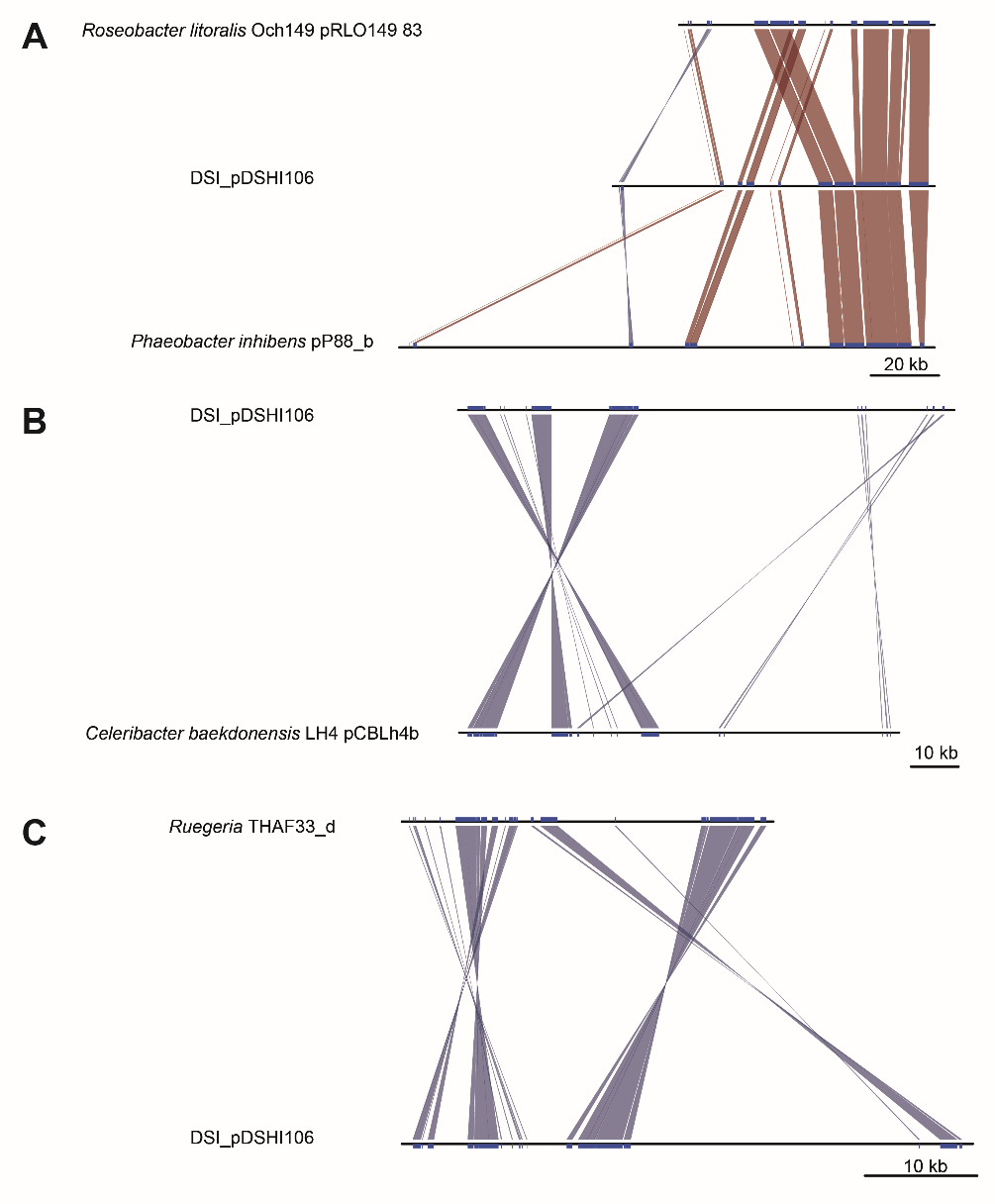


Figure S4B: Mauve alignment of the 102 kb plasmid (Dshi-6) with four Roseobacteraceae. Plasmid pP88_b of Phaeobacter inhibens, pRLO149_83 of Roseobacter litoralis Och 149, pTHAF33_d of Ruegeria plasmid and pCBLh4b of Celeribacter baekdonensis LH4 were used for the alignment.
