# Supplementary material for: The Sixth Element: a 102-kb RepABC Plasmid of Xenologous Origin Modulates Chromosomal Gene Expression in Dinoroseobacter shibae
Source: mSystems. 2022 Aug 3;7(4):e00264-22. doi: 10.1128/msystems.00264-22 (PMC9426580; doi:10.1128/msystems.00264-22)
Supplement: FIG S5 [file msystems.00264-22-s0005.docx]

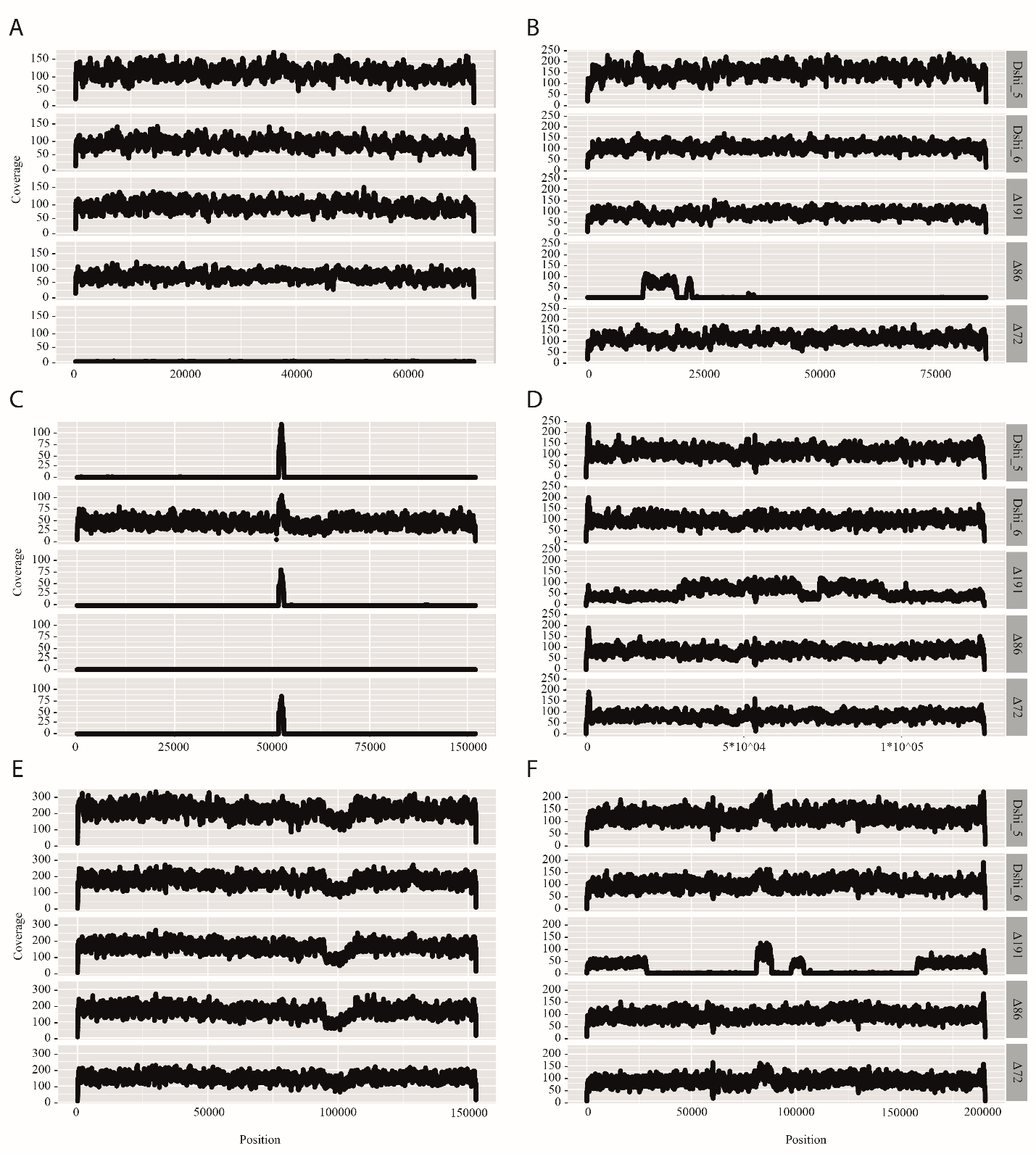


Figure S5A: Mapping of Illumina genome sequence reads on six ECRs of five Dinoroseobacter shibae strains investigated in the current study (Dshi-5, Dshi-6, Dshi-5Δ191, Dshi-5Δ86, Dshi-5Δ72). A) Δ72 kb, B) Δ86 kb, C) +102 kb, D) Δ126 kb, E) Δ153 kb, F) Δ191 kb.


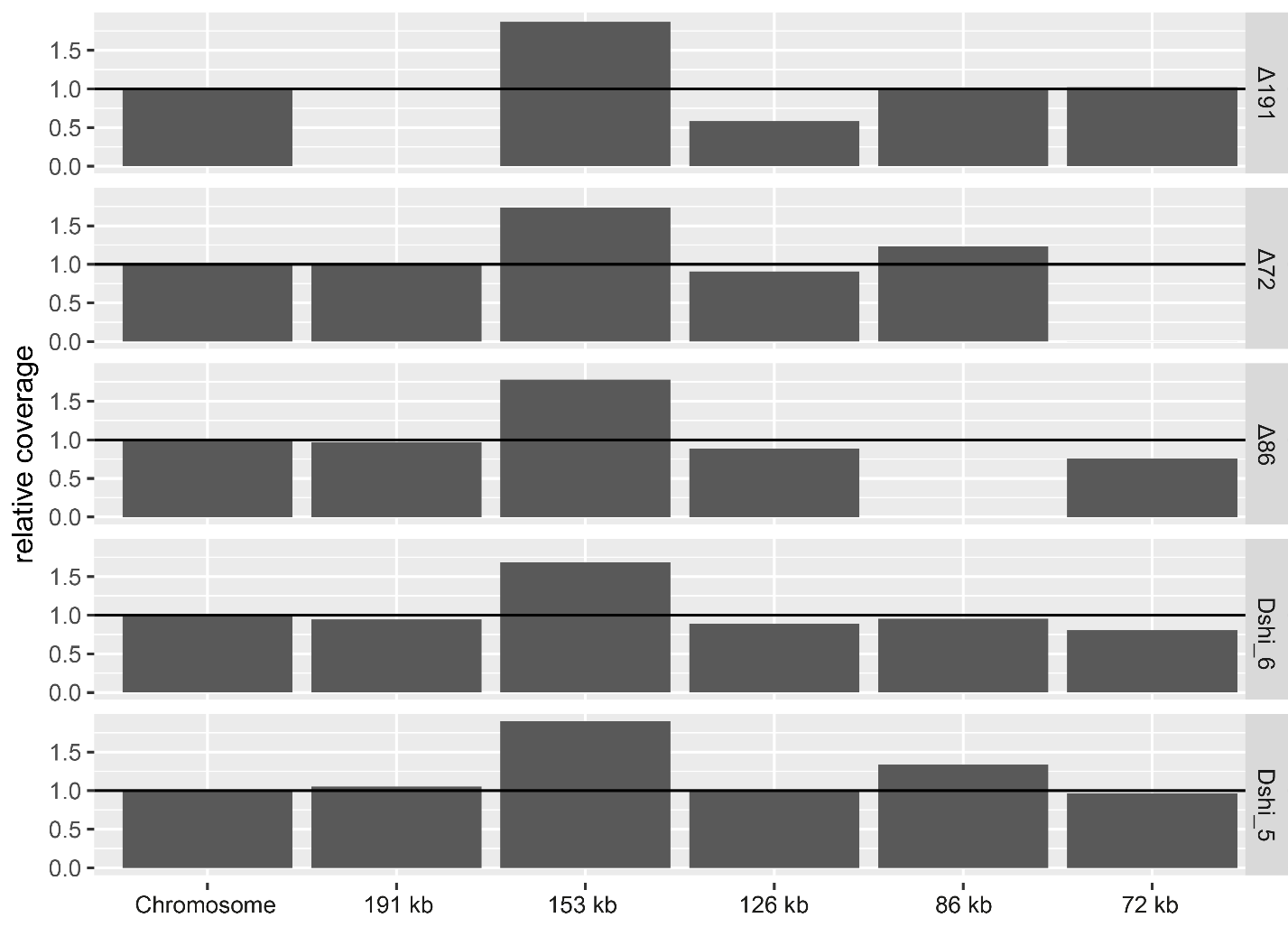


Figure S5B: Coverage of D. shibae replicons in different wild type and ECR-cured strains.
