# Supplementary material for: The Sixth Element: a 102-kb RepABC Plasmid of Xenologous Origin Modulates Chromosomal Gene Expression in Dinoroseobacter shibae
Source: mSystems. 2022 Aug 3;7(4):e00264-22. doi: 10.1128/msystems.00264-22 (PMC9426580; doi:10.1128/msystems.00264-22)
Supplement: FIG S6 [file msystems.00264-22-s0006.docx]

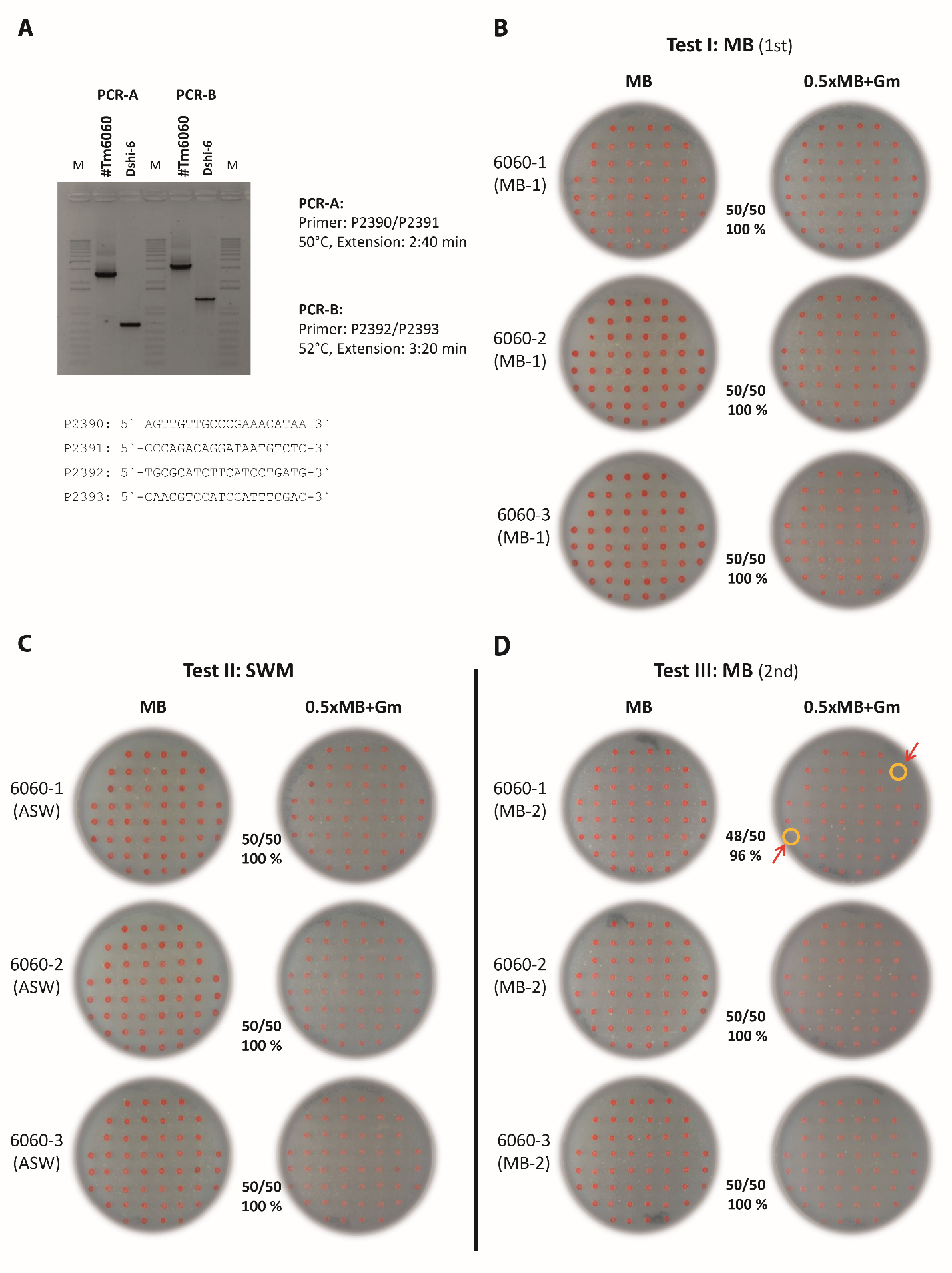


Figure S6: Transposon mutant and stability tests of the 102 kb strain. A) PCR-based validation of the D. shibae DSM 112351 transposon mutant #Tm6060. The mariner transposon with the gentamicin resistance gene was integrated into the 102 kb plasmid (Dshi-6::Gm_102kb; target gene: Dshi_6060). The wild type Dshi-6 served as a reference. M, marker (1 kb Plus Ladder Invitrogen). B and C) Stability test of the 102 kb D. shibae plasmid in MB and SWM medium with three replicates (Gm-tagged transposon mutant #Tm6060). 4 ml of the main culture were incubated with 40 µl preculture for ten days. D) Stability test of the 102 kb D. shibae plasmid in MB medium with three replicates (Gm-tagged transposon mutant #Tm6060). 4 ml of the main culture were incubated with an inoculation loop of cells grown on 0.5x MB with gentamicin and grown for seven days. Plasmid losses are indicated by a red arrow.
