# Supplementary material for: The Sixth Element: a 102-kb RepABC Plasmid of Xenologous Origin Modulates Chromosomal Gene Expression in Dinoroseobacter shibae
Source: mSystems. 2022 Aug 3;7(4):e00264-22. doi: 10.1128/msystems.00264-22 (PMC9426580; doi:10.1128/msystems.00264-22)
Supplement: FIG S7 [file msystems.00264-22-s0007.docx]

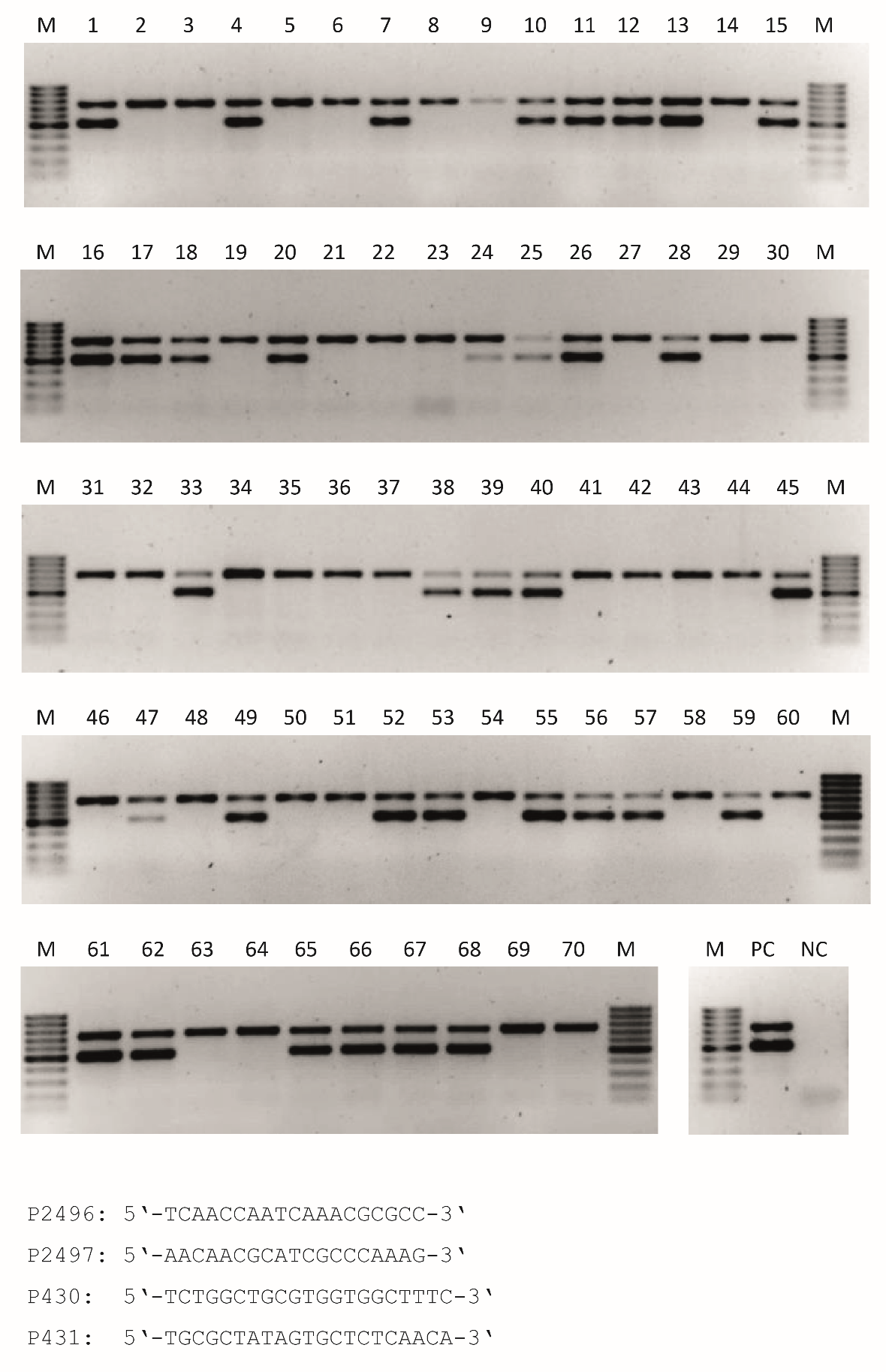


Figure S7: Duplex PCR of 70 D. shibae DSM 112351 (Dshi-6) colonies for the detection of the 102 kb plasmid (lower band, 518 bp; P2496/P2497) and the 191 kb plasmid (uper band, 730 bp; P430/P431). M, marker (1 kb Plus DNA Ladder Invitrogen); PC, positive control (isolated DNA from Dshi-6); NC, negative control.
