# Supplementary material for: The Sixth Element: a 102-kb RepABC Plasmid of Xenologous Origin Modulates Chromosomal Gene Expression in Dinoroseobacter shibae
Source: mSystems. 2022 Aug 3;7(4):e00264-22. doi: 10.1128/msystems.00264-22 (PMC9426580; doi:10.1128/msystems.00264-22)
Supplement: FIG S8 [file msystems.00264-22-s0008.docx]

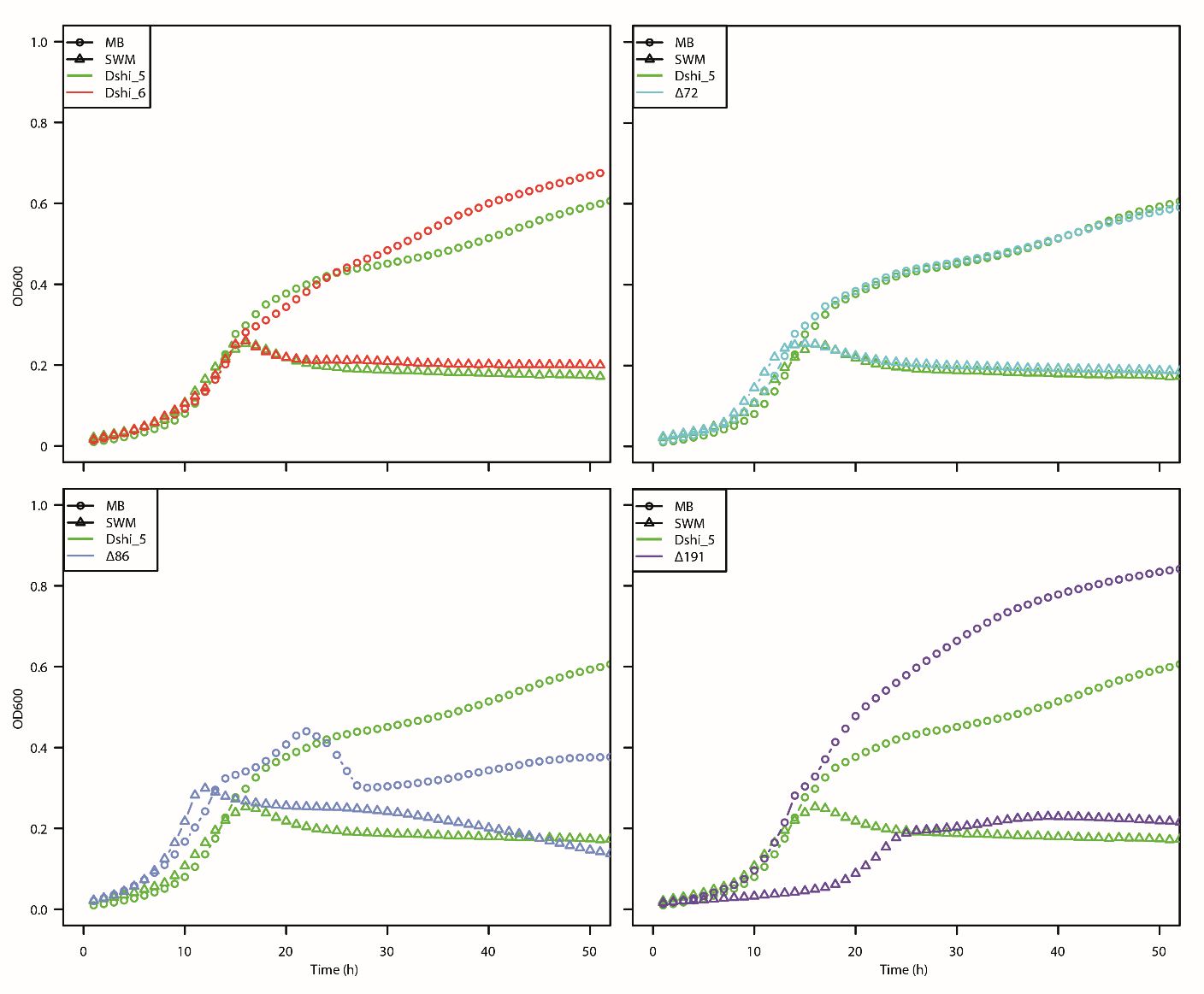


Figure S8: Growth kinetics of strains Dshi-5 (five ECRs), Dshi-6 (six ECRs) and three curing mutants of Dinoroseobacter shibae (Δ72, Δ86, Δ191; four ECRs) in different media. Strains were either grown in marine broth (MB) or artificial seawater (ASW) medium with succinate. Growth was monitored over 50h and measured at an optical density of 600 nm.
