# Supplementary material for: The Sixth Element: a 102-kb RepABC Plasmid of Xenologous Origin Modulates Chromosomal Gene Expression in Dinoroseobacter shibae
Source: mSystems. 2022 Aug 3;7(4):e00264-22. doi: 10.1128/msystems.00264-22 (PMC9426580; doi:10.1128/msystems.00264-22)
Supplement: FIG S9 [file msystems.00264-22-s0009.docx]

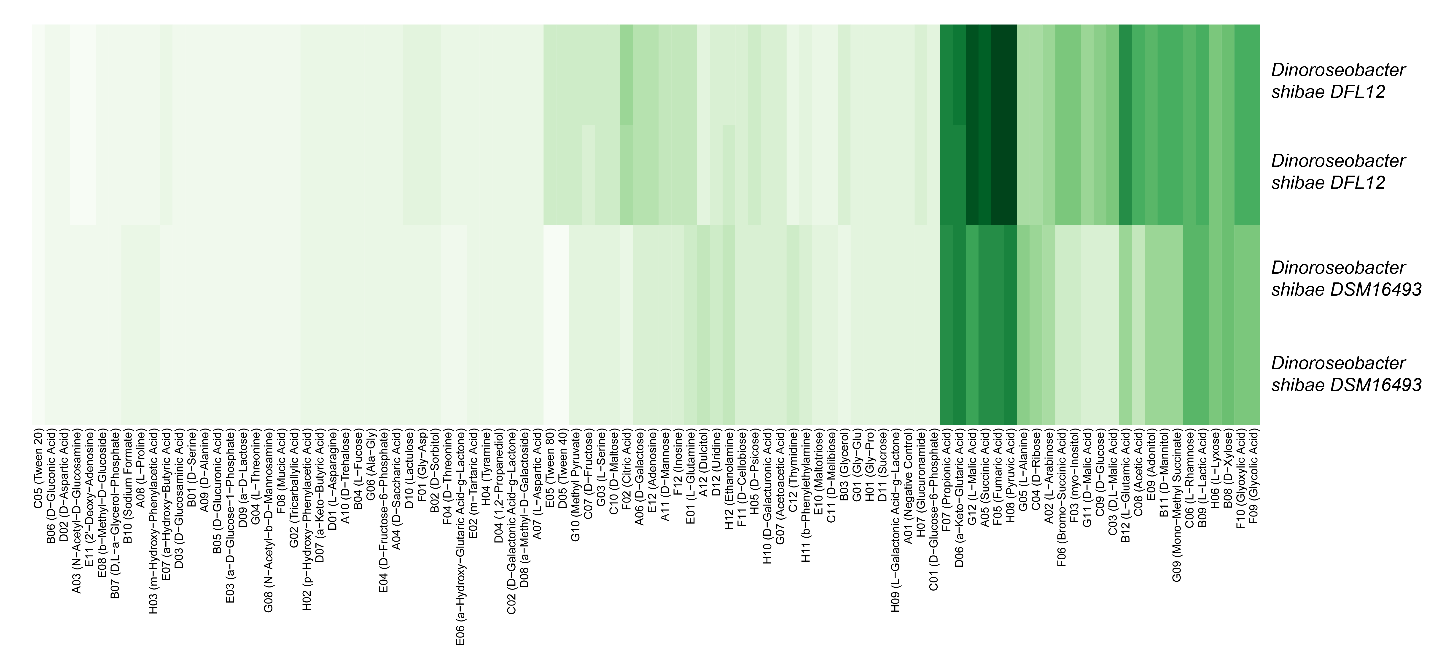


Dshi-5

Dshi-5

Dshi-6

Dshi-6

Figure S9A: Biolog Phenotypic Microarray (PM1) comparison showing the conversion of 95 carbon sources by Dshi-6 and Dshi-5 strains. Duplicates of each strain are shown.


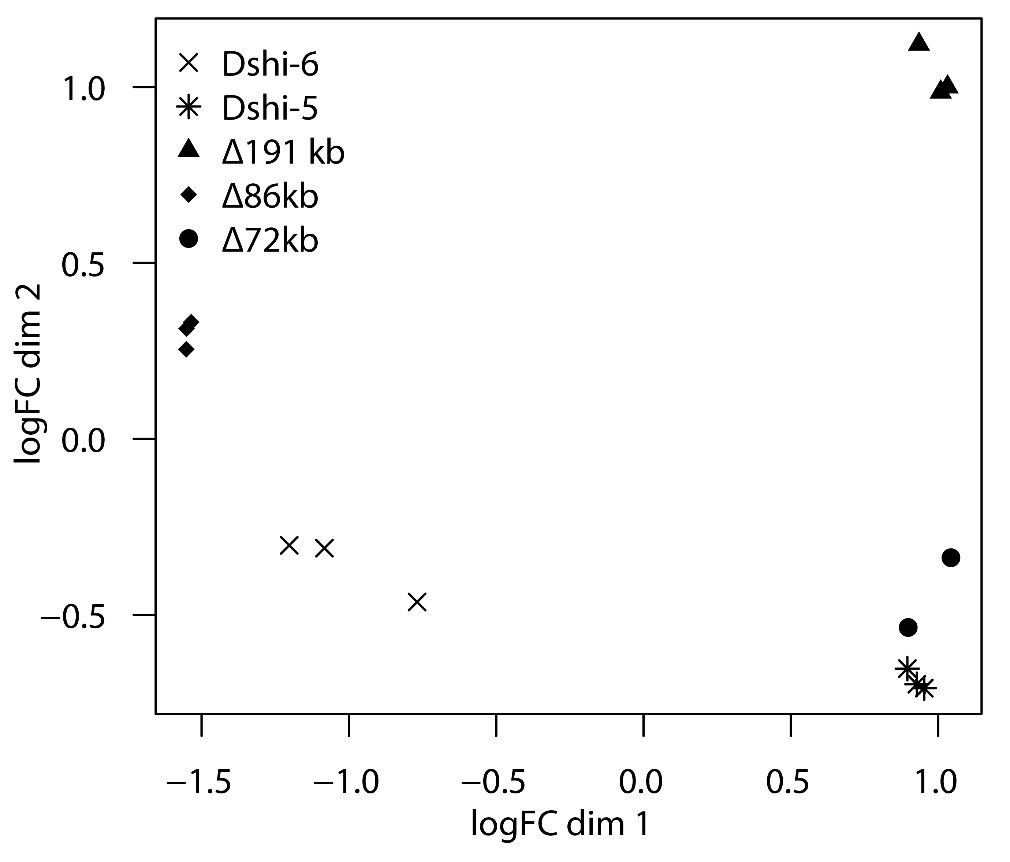


Figure S9B: Multidimensional scaling of the transcriptomic data sets analysed in this study.


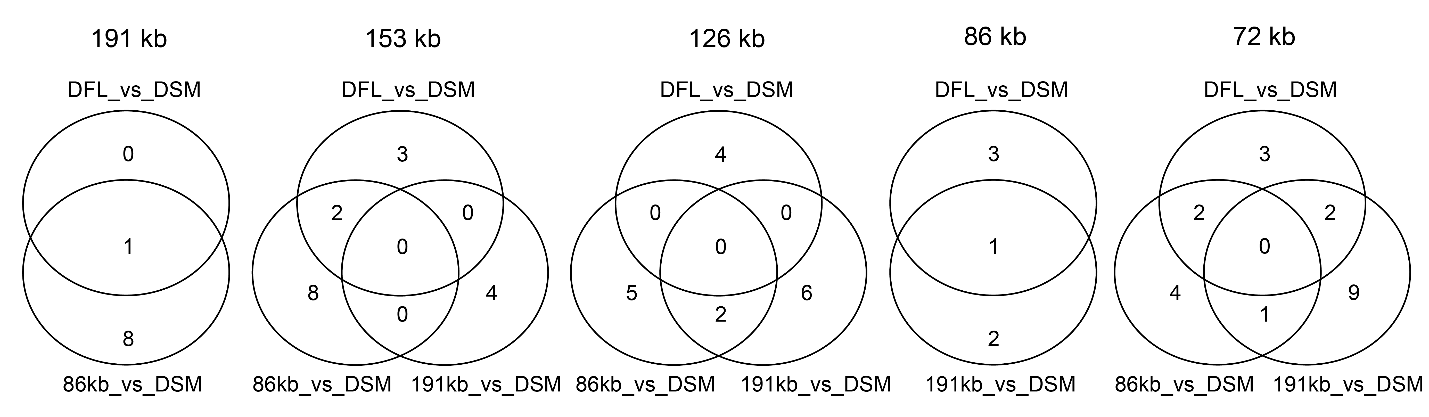


86 kb vs Dshi-5 191 kb vs Dshi-5

191 kb vs Dshi-5

86 kb vs Dshi-5 191 kb vs Dshi-5

86 kb vs Dshi-5 191 kb vs Dshi-5

86 kb vs Dshi-5

Dshi-6 vs Dshi-5

Dshi-6 vs Dshi-5

Dshi-6 vs Dshi-5

Dshi-6 vs Dshi-5

Dshi-6 vs Dshi-5

Figure S9C: Venn-diagram showing the number of extrachromosomal genes overlappingly regulated in each mutant strain. Dshi-5, Dshi-6.


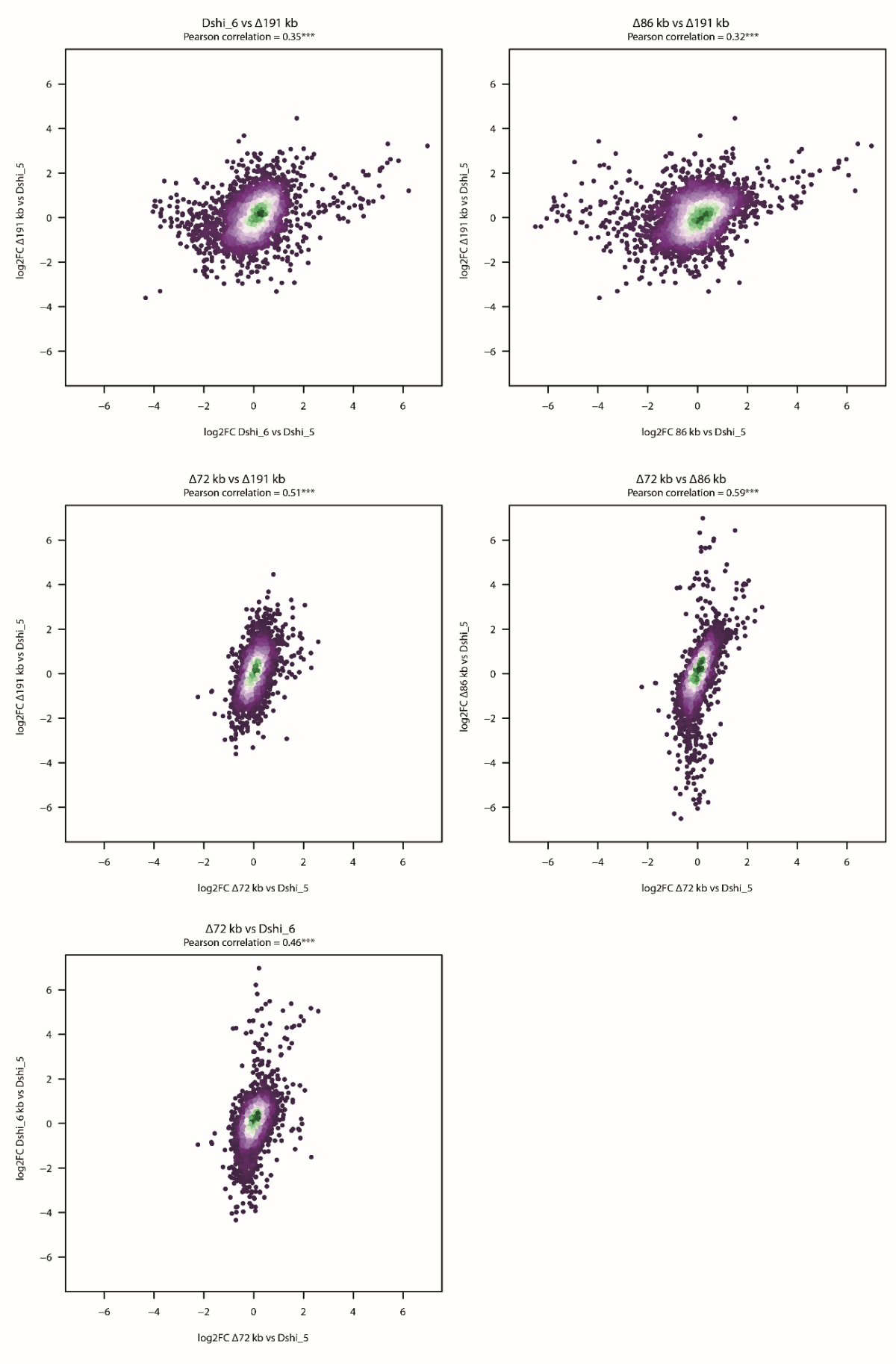


Figure S9D: Correlation of log2 fold change values of all genes in the different strains used in this study as compared to the reference strain Dshi-5. Pearson correlations are given.


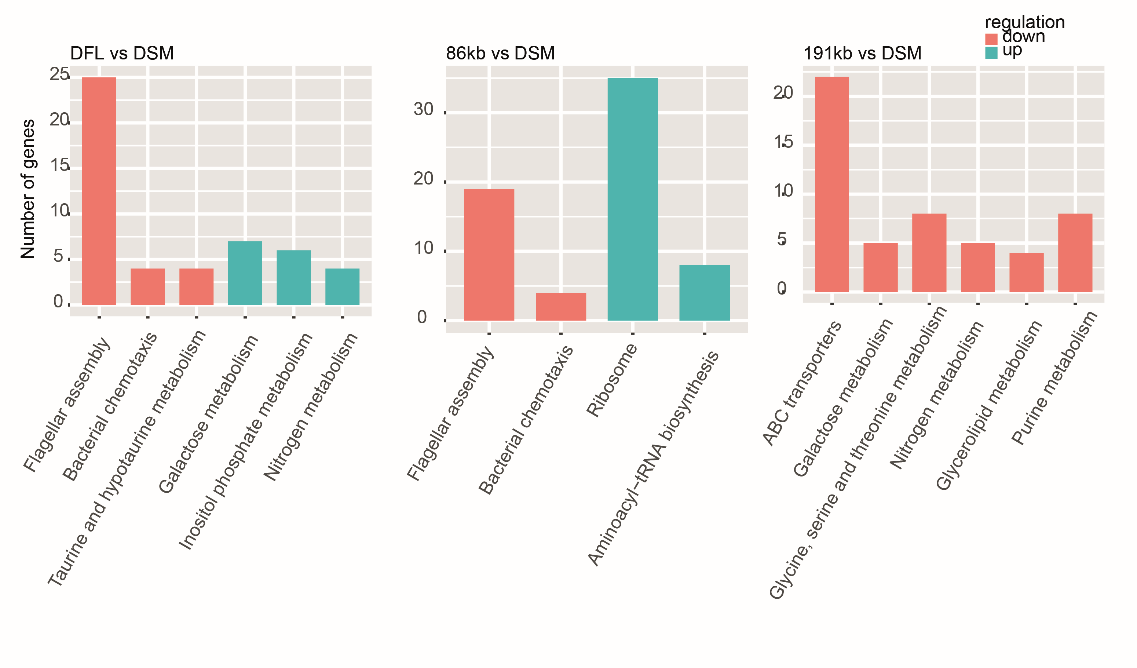


191 kb vs Dshi-5

86 kb vs Dshi-5

Dshi-6 vs Dshi-5

Figure S9E: Assessment of significantly differentially expressed genes in Dshi_6, 86 kb and 191 kb according to KEGG pathways.
